# Phytochemical Investigation and Biofilm-Inhibitory Activity of Bachtiari Savory (*Satureja bachtiarica* Bunge) Aerial Parts

**DOI:** 10.3390/plants13010067

**Published:** 2023-12-25

**Authors:** Marzieh Rahmani Samani, Gilda D’Urso, Filomena Nazzaro, Florinda Fratianni, Milena Masullo, Sonia Piacente

**Affiliations:** 1 Department of Pharmacy, University of Salerno, Via Giovanni II n. 132, 84084 Fisciano, Italy; mrahmanisamani@unisa.it (M.R.S.); gidurso@unisa.it (G.D.); mmasullo@unisa.it (M.M.); 2 PhD Program in Drug Discovery and Development, University of Salerno, via Giovanni Paolo II n. 132, 84084 Fisciano, Italy; 3 Institute of Food Science-National Research Council (CNR-ISA), Via Roma, 64, 83100 Avellino, Italy; filomena.nazzaro@isa.cnr.it (F.N.); fratianni@isa.cnr.it (F.F.)

**Keywords:** *Satureja bachtiarica* Bunge, LC-ESI/LTQOrbitrap/MS/MS^n^, NMR analysis, antioxidant activity, antibacterial activity, biofilm inhibition

## Abstract

*Satureja bachtiarica* is an endemic plant from the Lamiaceae family, growing in the Zagros mountain range in Iran. Even if *S. bachtiarica* is reported to possess many biological activities, little is known about its chemical composition. For this reason, in the present research, a phytochemical investigation of this species was carried out. To have a preliminary metabolite profile of *S. bachtiarica*, the *n*-BuOH extract was analyzed using LC-ESI/LTQOrbitrap/MS/MS in negative ion mode, allowing the identification of specialized metabolites belonging to flavonoid, monoterpene, indol, phenylpropanoid, phenolic, lignan, coumarin, biphenyl, and triterpene classes. The LC-MS/MS analysis guided the isolation of compounds, and their structures were characterized using spectroscopic methods including 1D- and 2D-NMR experiments and HRMS^n^ analysis. In this way, a compound never reported before belonging to the biphenyl class was identified. Total flavonoid content of the extract along with the antioxidant activity were assessed. Based on the traditional uses of *S. bachtiarica* suggesting potential antibacterial properties, an evaluation of the biofilm inhibitory activity of the extract and isolated compounds against mature biofilms of *Acinetobacter baumannii*, *Escherichia coli*, *Listeria monocytogenes*, *Pseudomonas aeruginosa*, and *Staphylococcus aureus*, as well as their influence on the metabolism of sessile bacterial cells, was conducted. The results evidenced that some compounds including parmentin B, biphenyls, and 1-(1H-indole-3-carboxylate)-β-D-glucopyranoside might inhibit some changes occurring in the bacterial cells, which increases their virulence. In particular, biphenyl derivatives at a concentration of 80 μg/mL were capable of limiting remarkably the mature biofilms of *A. baumannii* and *L. monocytogenes* remarkably at a percentage ranging between 52.76% and 75.02%, and they reached an inhibition percentage of 69.28 % against *E. coli*. Biphenyl derivatives were also effective in exerting an inhibitory action against the mature biofilm of *P. aeruginosa* (inhibition ranging from 59.38% to 81.08%) and *Staphylococcus aureus* (inhibition percentage reached 82.94%). Of note, the biphenyl derivatives resulted in being capable of acting on the metabolism of the cells within the biofilm of all five pathogens.

## 1. Introduction

The Lamiaceae family is one of the major sources of medicinal and food plants, highly valued mainly for their content in essential oils. Members of Lamiaceae find culinary applications as spices or are considered medicinal plants in Iranian folk medicine for their biological activities [1]. The essential oils of the Lamiaceae family have been studied extensively for several uses including antibacterial, antioxidant, and natural preservative properties [2].

The genus *Satureja* (Lamiaceae) was first named by the Roman writer Pliny. The name “Satureja” is derived from the Latin word “satureia”, and it means “herb of satyrs”. According to historical accounts, the cultivation of *Satureja* was banned in monasteries, possibly due to its association with pagan traditions or beliefs. *Satureja* comprises approximately 200 species of aromatic herbs and shrubs. These plants are known for their aromatic leaves and often have culinary and medicinal uses. More than 30 species of *Satureja* are found in the eastern parts of the Mediterranean area, where they are native [3]. Phytochemical investigations on *Satureja* spp. have revealed the occurrence of flavones, anthocyanins, phenolic acids, triterpenes, diterpenes, and sterols [4]. The essential oils derived from *Satureja* species have been found to exhibit a diverse array of biological and pharmacological properties, including antioxidant, anti-inflammatory, antispasmodic, analgesic, antiseptic, antibacterial, antiviral, and anti-nociceptive actions [1,5]. Moreover, the genus *Satureja* is receiving particular attention due to its antifungal and antimicrobial properties [6].

Bakhtiari savory (*S. bachtiarica*), also named “Marzeh e koohi” in Persian, has long been cultivated as a spice and as remedy in traditional medicine in Iran. The leaves are applied extensively as a flavoring agent for several type of foodstuffs due to their strong aroma and unique flavor [5,7]. The essential oil composition of the aerial parts of *S. bachtiarica* has been extensively investigated [5,8]. They represent a rich source of phenolic monoterpenes like carvacrol and thymol, along with other monoterpenes like γ-terpinene and *p*-cymene [9].

In the literature, few articles have reported on the biological properties of *S. bachtiarica* polar extracts. The ethanol extract from the *Satureja* species demonstrated an immunostimulant effect in Wistar rats [10], while the methanol extract was evaluated for its total flavonoid and phenolic content and antioxidant activity [7]. In a study by Alghooneh et al., the antibacterial and antioxidant capabilities of *S. bachtiarica* extract had a substantial impact on the characteristics of cheese [7]. In another study reported by Skendi et al., satureja, oregano, and thyme used in a bread recipe showed antimicrobial properties and could be used as antimicrobial ingredients in food products [6]. To the best of our knowledge, this is the first phytochemical investigation of the polar extract of the aerial parts of *S. bachtiarica* performed using HR-LC-ESI-Orbitrap-MS followed by isolation and structure elucidation of compounds via 1D- and 2D-NMR experiments. Moreover, to more deeply investigate the biological effects of *S. bachtiarica* extract, the total flavonoid content and the antioxidant activity of the extract were evaluated.

In recent years, there is an increasing interest in the identification of new antimicrobials to contrast the emergence of antibiotic resistance among pathogenic microorganisms, along with the formation of biofilm. Therefore, considering the reported activity of *Satureja* spp. including *S. bachtiarica* in foods, the antimicrobial activities of the extract and isolated compounds were evaluated against different Gram-positive and Gram-negative human bacteria. Their ability to inhibit the mature biofilms created by these bacteria and to affect the metabolism of microbial cells present within the biofilms was also tested.

## 2. Results and Discussion

### 2.1. LC-MS and NMR Analysis of Specialized Metabolites Occurring in S. bachtiarica n-BuOH Extract

**Figure 1:**
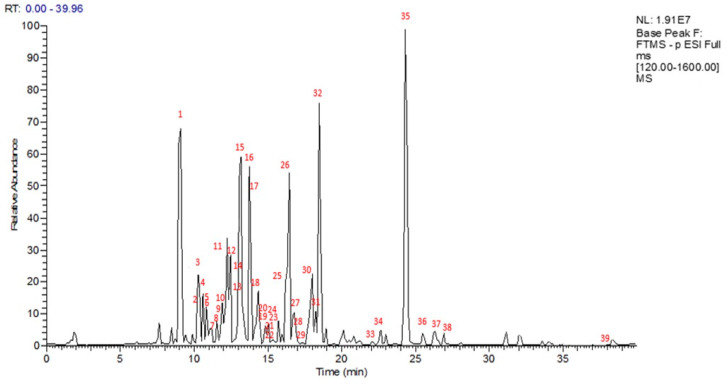
LC-MS profile in negative ion mode of *S. bachtiarica n*–BuOH extract.

To obtain a preliminary metabolite profile of *n*-BuOH extract of *S. bachtiarica*, an analytical approach based on LC-ESI-FT-MS and LC-ESI-FT-MS/MS analysis in negative ion mode was developed, with the detection of 39 compounds (Figure 1). Based on the accurate masses of the main peaks, their distinctive fragmentation patterns, and comparison of the results to information on the *Satureja* species found in the literature and database (“KNApSAcK”), some of the main peaks were putatively assigned (Table 1). To this extent, specialized metabolites of different classes, mainly corresponding to flavonoids (**1**, **6**–**14**, **19**, **20**, **22**–**23**, **25**–**26**, **27**, **30**–**34**), monoterpenes (**2**, **3**, **33**), indol (**4**), phenylpropanoids (**16**, **28**), phenolics (**5**, **15**, **17**, **18**, **29**), lignan (**21**), coumarin (**24**), biphenyls (**35**–**38**), and triterpene (**39**) were identified.

**Figure 2:**
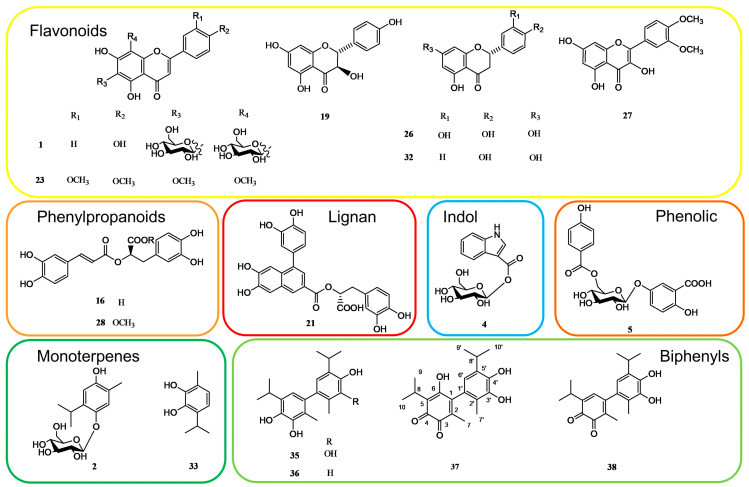
Some of the specialized metabolites isolated from *n*-BuOH extract of *S. bachtiarica*.

To obtain a comprehensive knowledge of the specialized metabolites of the aerial parts of *S. bachtiarica*, and to unambiguously attribute the peaks occurring in the LC-ESI-FT-MS/MS profile, a phytochemical investigation of the *n*-BuOH extract was performed. Thus, the *n*-BuOH extract was submitted to different chromatographic steps, using Sephadex LH-20 and HPLC-UV, affording compounds reported in Table 1. Their structures were elucidated using extensive spectroscopic methods including 1D- (1H, ^13^C, TOCSY) and 2D-NMR (DQF-COSY, HSQC and HMBC) experiments as well as ESI-MS analysis (Figure 2).

From the LC-MS profile, flavonoids resulted as one of the main phenolic classes in this species. The fragmentation pattern of compounds **1**, **6**–**14**, **19**–**20**, **22**–**23**, **25**–**27**, **30**–**32**, and **34** allowed identification of the presence of sugar units linked via different combinations to flavone (apigenin, luteolin, and diosmetin), flavonol (kaempferol and quercetin), flavanone (hesperidin, eriodictyol, and isosakuranetin), and flavanonol (dihydrokaempferol) aglycones giving rise to neutral losses of 162 and 132 Da corresponding to the loss of hexose and pentose units, respectively; a neutral loss of 120 Da corresponding to a C-linked hexose unit; and 176 Da related to a glucuronic unit. Compounds **25** and **31** were already reported in *S. bachtiarica* [1], while the other flavonoids are reported here for the first time in this species.

Compounds **5**, **15**–**18**, **28**, and **29** showed typical molecular formulae and fragmentation patterns observed for compounds belonging to the phenolic class. Compound **16** was one of the highest peaks in the LC-MS profile, and it was characterized as rosmarinic acid. Rosmarinic acid (**16**) and *p*-coumaric acid (**17**) were previously reported in *S. bachtiarica* [1]. Compounds **5**, **15**, **18**, **28**, and **29** were identified as parmentin B, 12-hydroxyjasmonic acid-(6-*O*-caffeoyl)-glucoside, (3R,7R)-tuberonic acid-12-*O*-[6′-*O*-(E)-feruloyl]-β-D-glucopyranoside, methylrosmarinic acid, and caffeic acid ethyl ester, respectively, reported here for the first time in *S. bachtiarica*.

Compounds **2**, **3**, and **33** were identified as monoterpenes. Compounds **2** and **3** showed the same [M-H]^−^ ion at *m*/*z* 327.1442, as well as the same molecular formula and fragmentation pattern with a loss of 162 Da (corresponding to sugar unit) [11]. They were identified as thymoquinol 5-*O*-β-D-glucopyranoside and thanol 3-*O*-β-D-glucoside, respectively, previously reported in the Lamiaceae family [12]. Compound **33** showed a [M-H]^−^ ion at *m*/*z* 165.0918 corresponding to the molecular formula C_10_H_14_O_2_ and two fragment ions at 151 and 138 Da; it was identified as thanol as previously reported in *Thymus vulgaris* leaves [13].

Compound **4** showed an ion at *m*/*z* 322.0925, supporting the molecular formula C_15_H_17_O_7_N, and it was identified as 1-(1H-Indole-3-carboxylate)-glucopyranose, reported in *Saccocalyx satureioides* [14]. Compound **21** was proposed as globoidnan A, which belongs to the lignan class; this was also confirmed by the fragment ion at *m*/*z* 311.06 which was similar to the fragmentation pattern already reported in the literature [15]. Compound **24** with a [M-H]^−^ ion at *m*/*z* 535.0872 and molecular formula C_27_H_20_O_12_ was identified as sagecoumarin, isolated previously from *Salvia officinalis* [16]. Compound **39** showed a [M-H]^−^ ion at *m*/*z* 455.3511 supporting a molecular formula C_30_H_48_O_3_, with fragment ions at *m*/*z* 411.33 [M-H-44]^−^, corresponding to the loss of CO_2_, and at *m*/*z* 409.34 and 343.29, corresponding to the fragmentation pattern reported in the literature for oleanolic acid, previously described in *S. montana* [17].

In the LC-MS profile, compounds **35**–**38** showed similar molecular formulae suggesting 20 carbon atoms. Their isolation and characterization via NMR analysis allowed us to identify these compounds as dimeric monoterpenes belonging to the biphenyl class.

In detail, compound **37** showed a [M-H]^−^ ion at *m*/*z* 343.1541, supporting a molecular formula C_20_H_24_O_5_; its MS/MS spectrum was characterized by three principal fragment ions at *m*/*z* 325.14 [M-H-18]^−^, 315.16 [M-H-28]^−^, and 300.10 [M-H-43]^−^, suggesting the presence of hydroxyl groups.

The ^1^H NMR spectrum showed the presence of two doublets at δ 1.26 and 1.22 (each 6H, *J* = 7.20 Hz) each coupled with benzylic septets at δ 3.29 and 3.25, respectively, indicating the presence of two isopropyl groups. The signals at δ 1.80 and 1.91 were attributed to two methyl groups linked to aromatic rings. In addition, only one aromatic singlet at δ 6.37 was observed in the ^1^H NMR spectrum. This pattern required a pentasubstituted phenyl moiety and a totally substituted phenyl moiety.

These observations were further supported by the ^13^C NMR spectrum, which showed twelve aromatic carbons, three of which hydroxylated (δ 147.0, 142.0, 142.0), six of which were quaternary (δ 146.0, 137.0, 124.0, 126.0, 120.8, 132.5), one of which was protonated (δ 118.3), and there were two signals at δ 185.0 and 186.7, supporting the presence of an ortho-quinone function. Other signals were attributed to two methine (δ 27.6, and 25.0) and six methyl carbons (δ 19.9 and 22.7, each for 2C and 12.5 13.2), which indicated the presence of two isopropyl and two methyl groups. Via a comparison of the NMR data (Table 2), it was possible to deduce for **37** a dimeric monoterpene structure giving rise to a biphenyl skeleton made up of a thanol moiety (**33**) and an orto thymoquinone-related moiety [18,19]. A detailed investigation suggested how the NMR data were comparable with those of 3′,4′-dihydroxy-5,5′-diisopropyl-2,2′-dimethylbiphenyl-3,4-dione (**38**), except for the absence of an aromatic proton. The HMBC spectrum showed for the quinone derivative the correlations between the methyl signal at δ 1.80 (Me-7) with the carbon resonances at δ 185.0 (C-3), 146.0 (C-1), and 137.0 (C-2), and between the proton signal at δ 3.25 (H-8) with the carbon resonances at δ 186.7 (C-4) and 147.0 (C-6). For the thanol moiety, correlations between the methyl signal at δ 1.91 (Me-7′) with the carbon resonances at δ 142.0 (C-3′), 126.0 (C-1′), and 120.8 (C-2′) and between the proton signal at δ 3.29 (H-8′) with the carbon resonances at δ 142.0 (C-4′) and 118.3 (C-6′) were observed in the HMBC spectrum. Furthermore, correlations between the proton signal at δ 6.37 (H-6′) with the carbon resonances at δ 146.0 (C-1), 142.0 (C-3′), 120.8 (C-2′), and 27.6 (C-8′) allowed us to establish the linkage between the thanol moiety and the orto thymoquinone-related moiety. Based on this evidence, the structure of compound **37**, never described previously, was assigned as reported in Figure 2 and named 6,3′,4′-trihydroxy-5,5′-diisopropyl-2,2′-dimethylbiphenyl-3,4-dione.

Compounds **35** and **36** were identified as 3,4,3′,4′-tetrahydroxy-5,5′-diisopropyl-2,2′-dimethylbiphenyl and 3,4,4′-trihydroxy-5,5′-diisopropyl-2,2′-dimethylbiphenyl [19,20].

The supposed reaction pathway for thanol (**33**), also known as p-cymene-2,3-diol, leading to the formation of the oxidation products **35**–**38** was previously described by Rainis for the dimeric oxidation products of thanol. It follows a common reaction pathway of phenols, which starts with oxygen-catalyzed radical formation of the monoterpene molecules via loss of two hydrogens (one from each molecule) and then proceeds with electron migration to the unsubstituted para carbon atom, leading to a radical dimerization and subsequently to the biphenyl compounds [19]. Biphenyl compounds were isolated from the leaves of thyme and showed to have deodorizing, antioxidative, and human platelet aggregation-inhibiting activities [18,19].

### 2.2. Evaluation of the Flavonoid Content and Antioxidant Activity of S. bachtiarica Extract

The total flavonoid content and the antioxidant activity of *S. bachtiarica* BuOH extract have been evaluated using the allumine chloride colorimetric assay and DPPH assay, respectively (Table 3). The DPPH assay revealed that the antiradical activity of the extract was 85.31 μg/mL, using ascorbic acid as a reference compound (1.35 μg/mL). This result agrees with the high content of flavonoids (162.33 μg/g of plant extract), expressed as rutin equivalents. The results suggest that the *S. bachtiarica* extract has significant antioxidant activity, which is likely attributed to its high flavonoid content.

### 2.3. Evaluation of the Antimicrobial Activity of Isolated Compounds

The minimal inhibitory concentration (MIC) exhibited by *S. bachtiarica* extract and isolated compounds was evaluated. The obtained results showed MIC equal or superior to 100 µg/mL. Two concentrations, 40 and 80 µg/mL, were used both in the crystal violet and in the MTT assays to have a scenario of the strength of each sample in inhibiting the mature biofilm and the metabolism of the sessile cells of five pathogenic strains. Results are shown in Table 4 and Table 5, respectively.

The inhibitory activity exhibited by samples against the mature biofilm was variegated depending on the strain (Table 4). *A. baumannii* was sensitive to the action of several compounds, particularly to some compounds like the flavonoid naringenin (**32**), to a monoterpene (**2**), and above all to biphenyl compounds (**35**–**38**), which caused an inhibition of the mature biofilm ranging between 52.76% and 74.21%. This confirms that, in addition to the presence of the most well-known molecules, such as flavonoids, phenolic acids, and lignans, biphenyls can play a significant inhibitory biofilm role against various pathogenic strains [21] including *A. baumannii* [22]. Moreover, globoidnan A (**21**) was determined to inhibit the mature biofilm of *A. baumannii* equal to 59.04%. To our knowledge, it is the first time that such a capacity has been observed for this lignan, which also exhibited a similar ability against all the other pathogenic strains used in our tests, Gram-positive and Gram-negative; therefore, although the results should be corroborated by further tests conducted on different pathogenic strains, we could hypothesize that this molecule potentially has a broad spectrum of action. The fact that the extract as a whole exhibited a biofilm inhibitory capacity that did not exceed 48%, compared to an inhibition % that even exceeded 74%, could be due to the presence of some compounds, in particular compounds **16**, **19**, **27**, and **28** (and to a lesser extent to compounds **1** and **4**), which not only proved ineffective against *A. baumannii* but also affected the inhibitory efficacy of the extract, somehow slowing down its inhibitory action.

The compounds 1-(1H-indole-3-carboxylate)-β-D-glucopyranoside (**4**) and parmentin B (**5**) were more effective in exerting an inhibitory action against the mature biofilm of *E. coli* (inhibition equal to 78.49% and 79.29%, respectively). For the first time, an excellent antimicrobial activity against *E. coli* has been highlighted for parmentin B. Of note, parmentin B (**5**) was isolated for the first time from *Parmentina cereifera* [23], a plant species whose extracts have shown various biological properties, including cardioprotective activity and antimicrobial activities (against *E.coli*, *S. aureus*, and *P. aeruginosa*), the latter determined using the inhibition zone test [24].

Furthermore, the biphenyl compounds (**35**–**38**) exhibited inhibitory biofilm activity, which reached an inhibition percentage of 69.28%.

Vicenin 2 (**1**), parmentin B (**5**), globoidnan A (**21**), and mainly biphenyl derivatives **35**–**38** proved to act effectively on the mature biofilm of *L. monocytogenes*, with an inhibition % ranging between 54.99% and 75.02%.

As regards the mature biofilm of *P. aeruginosa*, the 3,4,4′-trihydroxy-5,5′-diisopropyl-2,2′-dimethylbiphenyl (**36**) showed an excellent biofilm-inhibitory activity (above 80%). Also, the biphenyl derivatives **35** and **38** were able to exert a significant inhibitory action with values ranging from 64.54% to 68.94%. In the tests carried out on *P. aeruginosa*, the weakness of some compounds (**1**, **16**, **19**, and **27**) in inhibiting the biofilm of this pathogen could negatively affect the biofilm inhibitory action exhibited by extract. This trend was more evident against *S. aureus*, despite the very high inhibition values recorded for compounds **32** and **36**–**38** (Table 4).

The excellent inhibitory activity exhibited by biphenyl derivatives against all five pathogens used as tester strains confirms the antimicrobial and antibiofilm properties of biphenyls, which could be used against several Gram-positive and Gram-negative bacteria, in agreement with the literature data [25]. Indeed, some biphenyls were synthesized to enhance biological properties as the antimicrobial activity [26].

The antibiofilm activity exhibited by naringenin (**32**) against *S. aureus* expands the field of action regarding the use of such flavonoids against some of the most dangerous pathogens. Its effect can even improve the antibiofilm capacity of conventional antibacterial drugs [27].

Considering the chemistry of the tested compounds, among the flavonoid derivatives, compounds **1** and **23** showed higher inhibitory activities, while among the lignans, compound **21** showed the highest inhibitory activity.

A recent investigation tested the antimicrobial activity of rosmarinic acid (**16**) and globoidnan A (**21**) against *S. aureus* and *P. aeruginosa*, reporting MIC values higher than 300 µL/mL [28]. Herein, even if rosmarinic acid (**16**) was found not active, globoidnan A (**21**) showed interesting activity on the mature bacterial biofilms of these two strains.

Based on the above results, biphenyls **35**–**38**, dimeric oxidation products of thanol (**33**), showed a biofilm inhibitory activity higher than their precursor (**33**), especially against *A. baumannii*, *P. aeruginosa*, and *S. aureus*, while their effects against *E. coli* and *L. monocytogenes* were comparable to those of thanol.

The MTT test, performed to evaluate the capacity of the extract of *S. bachtiarica* and the individual compounds on the metabolism of the sessile cells, highlighted some interesting results (Table 5).

The data obtained against *A. baumannii* showed that the metabolism of its sessile cells could be inhibited by several compounds, compared to what was observed in the crystal violet test. The influence of the extract on the sessile metabolism of *A. baumannii* was undoubtedly more remarkable than in the CV test (inhibition = 67.35% and 47.99%, respectively). This data could imply that the inhibitory action would be very much expressed in its cellular metabolism and involved different compounds including flavonoids, monoterpenes, phenolic acids, and biphenyls; these last were not dominant, in contrast to the data from the violet crystal test. Most of the compounds determined an inhibitory action ranging between 49% and 68% with the highest activity shown by compounds **2**, **5**, and **32** (77.03%, 75.91%, and 70.71%, respectively). Once again, the inhibitory action on a pathogen could also be expressed by blocking or limiting the metabolic changes it undergoes that lead to an increase in virulence and greater resistance to conventional drugs.

The inhibitory influence on the metabolism of *E. coli* involved fewer compounds than what was observed against *A. baumannii*. Compound **19** showed that it inhibited the metabolism of *E. coli* (55.56%), along with compounds **27** (52.60%) and **28** (57.03). Compound **32**, which was determined to have an inhibition of 45.93% on the mature biofilm, gave the impression of acting above all on its cellular metabolism, as seen from the inhibition percentage data (72.93%). The results of the MTT test also displayed that compound **36**, which was determined to have an effective biofilm inhibitory action, also acted on the metabolism of *E. coli*, as demonstrated by the inhibition percentage (61.83%); similarly, compound **38** not only worked against the mature biofilm of *E. coli* but also gave higher inhibition values in the MTT test (61.47%). However, *S. bachtiarica* extract, overall, failed to act decisively on the metabolism of *E. coli*; in fact, while an inhibition percentage of 40.69% was recorded in the test with crystal violet (Table 4), in the MTT test we observed an inhibitory percentage of just 28.72%.

Compound **19** also inhibited the metabolism of *S. aureus* (inhibition = 42.99%). The results of the MTT test showed how the biphenyl derivatives **36**–**38**, which were determined to have an effective biofilm inhibitory action, also acted on the metabolism of *E. coli*.

Compounds **4**, **5**, **19**, **33**, and **36**–**39** were the most effective in inhibiting the metabolism of sessile cells of *L. monocytogenes*. This effectiveness would be associated with an almost equally effective response of *S. bachtiarica* extract, which determined an inhibition of the metabolism of *L. monocytogenes* equal to 55.32%.

Compounds as **23**, **32**, and **36**–**38** influenced the sessile cell metabolism of *P. aeruginosa* in a percentage ranging from 42.39% to 58.92%, even if a weaker inhibitory action was observed for the extract.

In the test conducted against *S. aureus*, compounds **1**, **4**, **5**, **19**, **33**, and **35**–**38** were the most active in counteracting the metabolism of the sessile cells in the range 46.49–68.93%.

The behavior exhibited by compounds regarding how they affected the metabolism of the cells present within the biofilm in some cases differed with respect to the inhibitory effect on the biofilm formation. Thus, compounds **1**, **4**, **5**, **16**, **19**, **21**, **27**, and **28**, although demonstrating less or no strength in inhibiting the formation of biofilm of *A. baumannii*, were more effective in inhibiting the metabolism of sessile cells once the biofilm was formed.

## 3. Experimental Setup

### 3.1. General Methods

NMR experiments were performed in methanol-d_4_ (99.95%, Sigma-Aldrich, Milan, Italy) on a Bruker DRX600 spectrometer (Bruker BioSpin GmBH, Rheinstetten, Germany) equipped with a Bruker 5 mm PATXI Probe at 300 K. Topspin 3.2 software was used to process the data. Semi-preparative HPLC-UV was performed with Phenomenex C18 Synergy-Hydro-RP (25 cm × 10 mm, 10 μm) on an Agilent 1260 Infinity system (Agilent Technologies, Palo Alto, CA, USA), equipped with a binary pump (G-1312C), a UV detector (G-1314 B), and a Rheodyne injector with a loop of 20 µL. TLC (Thin Layer Chromatography) was performed on silica gel F_254_ (Macherey-Nagel Deltek, Naples, Italy) plates (20 cm × 10 cm), and mixtures of CHCl_3_–CH_3_OH–H_2_O (80:18:2), BuOH, acetic acid, and H_2_O (60:15:25) (VWR international PBI s.r.l., Milan, Italy) were used as mobile phases to obtain a separate ion distance of 80 mm. Detection was carried out by spraying cerium (IV) sulphate followed by heating at 100 °C for five minutes. HR/ESI-MS data were acquired on an LTQ Orbitrap XL mass spectrometer (Thermo Fisher Scientific, San Josè, CA, USA) operating in negative ion mode.

### 3.2. Reagents

Ethanol and water for extraction and HPLC grade solvents were purchased from VWR International PBI (Milan, Italy). LC-MS grade solvents were procured by Merck (Darmstadt, Germany). DPPH (2,2-Diphenyl-1-picrylhydrazyl), NaOH (sodium hydroxide), NaNO_2_ (sodium nitrite), AlCl_3_ (aluminum chloride), and methanol-*d*_4_ were purchased from Sigma-Aldrich (Darmstadt, Germany).

### 3.3. Plant Material

The aerial parts of *S. bachtiarica* Bunge were collected in Chaharmahal va Bakhtiari, southern Iran, before flowering, 2400 m above sea level in July 2019 at 32°17′ N and 50°33′ E. The identification of *S. bachtiarica* Bunge was confirmed by Dr. Shirmardi, H.A., Ph.D. (Research Center of Agriculture and Natural Resources, Shahrekord, Iran), and voucher specimens were deposited in the Herbarium of this Research Center (No. 3621). The plants were consequently air dried for five days at 30 ± 2 °C in a shaded environment.

### 3.4. Extraction

Air-dried and powdered aerial parts of *S. bachtiarica* Bunge (50 g) were extracted with ethanol/water (70: 30, *v*/*v*) (3 × 3 L, 72 h) at room temperature. The dried ethanol/water extract (5.17 g) was obtained after filtration and solvent evaporation to dryness in a vacuum. The residue was dissolved in water and extracted with *n*-BuOH (40 mL) to remove free sugars. After filtration and solvent evaporation to dryness under reduced pressure, 3.16 g of the *n*-BuOH extract were obtained.

### 3.5. HR-LC-ESI-Orbitrap-MS and HR-LC-ESI-Orbitrap-MS/MS Analysis

*S. bachtiarica n*-BuOH extract was analyzed by LC-ESI/LTQOrbitrap/MS using a HPLC coupled to a hybrid mass spectrometer, which combines the linear trap quadrupole (LTQ) and Orbitrap mass analyzer in negative ion mode. All experiments were performed using a Thermo scientific liquid chromatography system constituted of a quaternary Accela 600 pump and an Accela autosampler, connected to an LTQ-Orbitrap XL mass spectrometer with electrospray ionization (ESI). An amount of 5 µL of the sample was injected with the mass spectrometer operating in negative ion mode with a mobile phase consisting of H_2_O + 0.1% formic acid (A) and CH_3_CN + 0.1% formic acid (B) and a stationary phase consisting of Luna C18 5 μm (150 × 2.1 mm) (Phenomenex, Aschaffenburg, Germany) column. A linear gradient held at a flow rate of 0.200 mL/min of solvent B was used: 0–35 min, from 5 to 95%; 35–40 min, isocratic at 95%; then, back to 5% for 10 min. ESI source parameters were as follows: capillary voltage—48 V; tube lens voltage—176.47; capillary temperature 280 °C; sheath gas flow at 15 (arbitrary units); auxiliary gas flow at 5 (arbitrary units); sweep gas 0; and spray voltage 5. Full range acquisition was set at *m*/*z* 120–1600, while for the MS/MS study, a data-dependent scan was carried out, selecting precursor ions corresponding to the most intensive peaks in the LC-MS analysis. Xcalibur software version 2.1 was used for instrument control, data acquisition, and data analysis.

### 3.6. Extraction and Isolation Procedure

An amount of 3 g of *n*-BuOH extract was fractionated on a Sephadex LH-20 (GE Healthcare, Sigma Aldrich, Milan, Italy) column (100 × 5 cm), using MeOH as the mobile phase, affording 82 fractions (10 mL), as monitored using TLC. Based on TLC analysis, fractions with similar compositions were combined to yield 14 main fractions. Further purifications were carried out using an RP-HPLC-UV system. Fractions were dissolved in MeOH in a concentration of 100 mg/mL with injections of 20 μL for a total of 14 chromatographic runs. The elution gradient was obtained using H_2_O + 0.1% formic acid (A) and CH_3_CN + 0.1% formic acid (B) as mobile phases at a flow rate of 2.0 mL/min. The detection wavelength was 254 nm, and the analysis was performed at room temperature. The peaks collected at HPLC-UV were evaporated to dryness in a vacuum and were dried before NMR analysis.

For fractions 5, 6, 7, and 11–14, the HPLC gradient started at 5% B; after 30 min % B was 100%, and it remained at 100% for 5 min. Fraction 5 (237.3 mg) afforded compounds 2 (3.9 mg, *t_R_* = 14.91 min), 15 (1.6 mg, *t_R_* = 16.55 min), and 18 (2.7 mg, *t_R_* = 17.67 min).

Compounds **33** (1.2 mg, *t_R_* = 25.70 min), **35** (2.4 mg, *t_R_* = 26.58 min), and **36** (1.4 mg, *t_R_* = 28.31 min) were isolated from fraction 6 (182.3 mg). Compounds **1** (1 mg, *t_R_* = 13.07 min), **4** (1.2 mg, *t_R_* = 14.82 min), **29** (1.4 mg, *t_R_* = 21.35 min), and **38** (1.6 mg, *t_R_* = 26.61 min) were isolated from fraction 7 (95.8 mg); compounds **16** (3.7 mg, *t_R_* = 17.38 min), **19** (1.2 mg, *t_R_* = 18.88 min), **28** (1.2 mg, *t_R_* = 20.05 min), and **34** (1.2 mg, *t_R_* = 25.68 min) were isolated from fraction 10 (94.6 mg); compounds **9** (1.2 mg, *t_R_* = 11.5 min), **27** (1.2 mg, *t_R_* = 20.95 min), and **32** (4.3 mg, *t_R_* = 21.80 min) were isolated from fraction 11 (107.6 mg); compounds **11** (1.2 mg, *t_R_* = 14.80 min), **7** (1.2 mg, *t_R_* = 15.50 min), **5** (1.3 mg, *t_R_* = 16.10 min), **14** (1.2 mg, *t_R_* = 16.70 min), and **26** (1.3 mg, *t_R_* = 20.25 min) were isolated from fraction 12 (29.4 mg); compounds **10** (1.2 mg, *t_R_* = 15.35 min), **8** (1.1 mg, *t_R_* = 13.01 min), **13** (1.2 mg, *t_R_* = 16.60 min), **24** (1.6 mg, *t_R_* = 18.62 min), and **31** (1.2 mg, *t_R_* = 21.29 min) were isolated from fraction 13 (53.4 mg); and compounds **21** (1.8 mg, *t_R_* = 18.03 min) and **25** (2.6 mg, *t_R_* = 20.08 min) were isolated fraction 14 (58.5 mg).

Fraction 6 was further purified using a linear gradient starting from 5% to 20% of B over 5 min, and later from 20% to 60% of B over 20 min, and from 60% to 85% of B over 5 min; later, the linear gradient went from 85% to 100% of B in 10 min, yielding compounds **3** (1.2 mg, *t_R_* = 22.95 min), **12** (1.3 mg, *t_R_* = 17.46 min), **37** (1.8 mg, *t_R_* = 35.53 min), and **39** (1.2 mg, *t_R_* = 48.40 min).

Fraction 8 (44.5 mg) was purified using a linear gradient starting from 5% to 36% of B over 15 min, then from 36% to 40% of B over 5 min, from 40% to 55% of B over 15 min, and after 5 min it reached 100% of B. Compounds **17** (1.2 mg, *t_R_* = 16.75 min) and **20** (1.2 mg, *t_R_* = 18.05 min) were obtained. Finally, fraction 9 (96.5 mg) was purified using a HPLC gradient starting from 5% to 52% of B over 10 min, then from 52% to 60% of B over 5 min, and from 60% to 70% of B over 20 min, and in 5 min 100% of B was reached. Compounds **6** (1.3 mg, *t_R_* = 12.80 min), **22** (1.2 mg, *t_R_* = 21.54 min), **23** (1.0 mg, *t_R_* = 23.92 min), and **30** (1.2 mg, *t_R_* = 19.04 min) were isolated.

### 3.7. Total Flavonoid Content

The previously published method was followed to quantify the total flavonoid content using the allumine chloride colorimetric assay and rutin as a reference (Supplementary Materials) [29].

### 3.8. DPPH^•^ Radical Scavenging Activity

For DPPH, the percentage of DPPH^•^ radical scavenging activity (%) was plotted against the extract concentration (µg/mL) to determine the IC_50_ (Supplementary Materials) [30].

### 3.9. Antimicrobial Activity

#### 3.9.1. Microorganisms and Culture Conditions

*Acinetobacter baumannii* (ATCC 19606), *Escherichia coli* (DSM 8579), *Pseudomonas aeruginosa* (DSM 50071), *Listeria monocytogenes* (ATCC 7644), and *Staphylococcus aureus* subsp. *aureus* Rosebach (ATCC 25923) were used as tester strains in the experiments. Before the tests, they were cultured in Luria Broth for at 37 °C or 35 °C (depending on the strain) for 18 h and 80 rpm (Corning LSE, Pisa, Italy).

#### 3.9.2. Minimal Inhibitory Concentration (MIC)

The MIC was evaluated using the resazurin microtiter-plate assay [31], in flat-bottomed 96-well microtiter plates (Falcon, VWR International, Milan, Italy), which were incubated at 37 °C or 35 °C for 24 h. The MIC value was revealed by the visual color change from dark purple to colorless. Tetracycline (previously dissolved in DMSO, 1 mg/mL) and sterile DMSO were the positive and negative controls, respectively. Determinations were performed in triplicate.

#### 3.9.3. Inhibition of Mature Biofilm

The capacity of *S. bachtiarica* extract and purified compounds to affect the mature bacterial biofilm was evaluated in flat-bottomed 96-well microtiter plates [32]. The overnight bacterial cultures were adjusted to 0.5 McFarland with fresh culture broth. Later, 10 µL of the bacterial cultures and sterile Luria–Bertani broth (LB, Sigma Aldrich Italia, Milan, Italy) were added to each well to reach a final volume of 250 µL. The microtiter plates were covered with parafilm tape to preclude the evaporation of material included in the wells and incubated for 24 h at 37 °C or 35 °C. Planktonic cells were removed; then, 40 or 80 μg/mL of the *Satureja* extract or singular compounds were added, and sterile Luria–Bertani broth was added to reach a final volume of 250 μL. After another 24 h of incubation at 37 °C or 35 °C, following the removal of the planktonic cells, the attached cells were lightly washed twice with sterile phosphate buffered saline (PBS), which was then discarded. The plates were kept for 10 min under a flow laminar hood before the addition of 200 µL of methanol in each well for 15 min to allow the fixation of the sessile cells. The methanol was then discarded, and each plate was left to dry; then, 200 µL of 2% *w*/*v* crystal violet solution were added to each well. After 20 min, the staining solution was removed, and the plates were lightly washed with sterile PBS and left to dry. The addition of 200 µL of glacial acetic acid 20% *w*/*v* allowed the release of the bound dye. The absorbance was measured at λ = 540 nm (Cary50Bio, Varian, Palo Alto, CA, USA). The percent value of adhesion was calculated with respect to the control (represented by the bacterial cells grown without the presence of the samples, for which the inhibition rate was assumed as 0%). Triplicate tests were performed, and results were expressed as the mean ± SD.

#### 3.9.4. Inhibitory Activity of Metabolism of Sessile Bacterial Cells

The effect of *S. bachtiarica* extracts and their compounds on the metabolic activity of sessile cells was evaluated using the 3-(4,5-dimethylthiazol-2-yl)-2,5-diphenyltetrazolium bromide (MTT) colorimetric method using 96-well microtiter plates [32]. The overnight bacterial cultures were adjusted to 0.5 McFarland, and the plates, with Luria broth up to 250 μL, were prepared as previously described. After 24 h total of incubation, bacterial suspension, representing the planktonic cells, was removed, and as previously described, 40 and 80 μL/mL of samples were added, along with Luria–Bertani broth, to reach a final volume of 250 mL. Following another 24 h of incubation, planktonic cells were removed, and 150 µL of PBS and 30 µL of 0.3% of MTT (Sigma, Milan, Italy) were added to each well, keeping microplates at 37 °C or 35 °C. The MTT solution was removed after two h, and the microplates were twice washed with 200 µL sterile physiological solution. Next, 200 µL of DMSO were added, leading to the formazan crystals’ dissolution, measured at 570 nm after two h. Triplicate tests were done, taking the average results for reproducibility, and results were expressed as the mean ± SD.

## 4. Conclusions

In the present study, a total of 39 compounds were detected in *S. bachtiarica n*-BuOH extract using a LC-ESI/LTQOrbitrap/MS/MS analysis. The LC-MS analysis guided the isolation of compounds, whose structures were elucidated using NMR analysis allowing us to identify specialized metabolites belonging to different classes. The phytochemical investigation revealed that some compounds such as rosmarinic acid (**16**), *p*-coumaric acid (**17**), luteolin (**25**), and apigenin (**31**) were already reported as present in the aerial parts of *S. bachtiarica* [3], while several other compounds (**1**–**15**, **18**–**19**, **21**–**24**, **26**–**30**, and **32**–**39**) are reported here for the first time in *S. bachtiarica*. Of note, the biphenyl derivative (**37**) is a molecule never reported before in the literature.

In this work, the antioxidant potential of *S. bachtiarica* extract was investigated, highlighting interesting antioxidant activity, related to phenolic and flavonoid content. Furthermore, interesting antimicrobial activity was observed for the tested compounds, which were able to inhibit the mature biofilms produced by different bacteria and to affect the metabolism of microbial cells present within the biofilms.

It is worth noting that some compounds exhibit excellent activity; these compounds include 1-(1H-indole-3-carboxylate)-β-D-glucopyranoside (**4**), parmentin B (**5**), dihydrokaempferol (**19**), and biphenyl derivatives (**35**–**38**), capable of remarkably limiting the mature biofilm and the metabolism of sessile cells of one or all five pathogens. Among the tested compounds, parmentin B (**5**) exerted an inhibitory action on the metabolism of the sessile cells of all five pathogenic strains, with a preference of action for the sessile cells of *A. baumannii*, *L. monocytogenes*, and *S. aureus*. Of note, the biphenyl derivatives **36**–**38** resulted in being capable of acting in a highly effective manner not only on the mature biofilm but also on the metabolism of the cells within the biofilm.

To our knowledge, this is the first time that such a high-performance efficacy of biphenyl compounds on the metabolism of sessile cells, which, therefore, as previously mentioned, are somehow “blocked” regarding the possibility of transforming thus becoming more virulent and more resistant to the actions of conventional antibiotics, has been demonstrated. The fact that these compounds act effectively against all five pathogenic strains assumes a particular relevance, considering the importance of these pathogens from a clinical and nutritional point of view.

Based on the obtained results, *S. bachtiarica* can be considered a rich source of bioactive compounds with antibacterial properties. These data reinforce the use of *S. bachtiarica* in human nutrition as a food rich in different classes of bioactive and healthy flavonoids and phenolics with beneficial effects.

## Figures and Tables

**Table 1:** Identified compounds in aerial parts of *S. bachtiarica* using LC-ESI/LTQOrbitrap/MS/MS^n^ analysis in negative ion mode and NMR analysis.

No.	Rt	[M-H]^−^	Molecular Formula	Δ ppm	MS/MS	Identity
**1**	8.95	593.1500	C_27_H_30_O_15_	−0.129	473.11/503.12/383.08/353.07	vicenin 2
**2**	10.12	327.1442	C_16_H_24_O_7_	1.133	165.09/229.14/291.20/211.13/171.10/309.21	thymoquinol 5-*O*-β-D-glucopyranoside
**3**	10.24	327.1444	C_16_H_24_O_7_	1.774	165.09/229.14/291.20/211.13/171.10/309.21	thanol 3-*O*-β-D-glucopyranoside
**4**	10.33	322.0925	C_15_H_16_O_7_N	1.24	262.07/160.04/202.05	1-(1H-indole-3-carboxylate)-β-D-glucopyranoside
**5**	10.73	435.0920	C_20_H_20_O_11_	−0.271	271.06/273.11/198.54/299.09	parmetin B
**6**	10.85	593.1500	C_27_H_30_O_15_	−0.011	285.04	luteolin 7-*O*-rutinoside
**7**	11.08	431.0970	C_21_H_20_O_10_	−0.587	311.06/269.05	apigenin 7-*O*-β-D-glucopyranoside
**8**	11.60	447.0922	C_21_H_20_O_11_	0.072	285.04	kaempferol 3-*O*-β-D-glucopyranoside
**9**	11.72	463.0871	C_21_H_20_O_12_	−0.610	301.03	quercetin-3-*O*-β-D-glucopyranoside
**10**	11.77	461.0714	C_21_H_18_O_12_	−0.027	285.05	luteolin 7-*O*-β-D-glucuronopyranoside
**11**	12.19	607.1661	C_28_H_32_O_15_	0.648	299.06/284.03	diosmin
**12**	12.47	609.1820	C_28_H_34_O_15_	1.105	301.07/310.05	hesperidin
**13**	12.94	417.0818	C_20_H_18_O_10_	0.616	285.04	luteolin 7-*O*-xyloside
**14**	13.01	445.0762	C_22_H_22_O_10_	−0.557	281.07	apigenin 4′-*O*-methyl 7-*O*-β-D-glucopyranoside
**15**	13.06	549.1963	C_27_H_34_O_12_	−0.551	387.17	12-hydroxyjasmonic acid (6′-*O*-caffeoyl)-β-D-glucopyranoside
**16**	13.74	359.0766	C_18_H_16_O_8_	1.437	161.02/179.03/197.05/223.02/133.03	rosmarinic acid
**17**	13.86	163.0401	C_9_H_8_O_3_	−1.0	145.02/119.05	*p*-coumaric acid
**18**	14.33	563.2123	C_28_H_36_O_12_	0.066	387.16/531.19/489.18	tuberonic acid-12-*O*-[6′-*O*-(E)-feruloyl]-*β*-D-glucopyranoside
**19**	14.80	287.0554	C_15_H_12_O_6_	1.622	259.06/243.07	dihydrokaempferol
**20**	14.88	593.1868	C_28_H_34_O_14_	0.58	285.08	isosakuranetin 7-*O*-rutinoside
**21**	15.03	491.0971	C_26_H_20_O_10_	−0.210	311.06	globoidnan A
**22**	15.26	343.0813	C_18_H_16_O_7_	0.46	328.25/313.20/285.08	eupatilin
**23**	15.50	373.0918	C_19_H_18_O_8_	0.17	327.14	hymenoxin
**24**	15.73	535.0872	C_27_H_20_O_12_	0.220	359.08/355.04/177.02/313.07/161.02	sagecoumarin
**25**	16.19	285.0398	C_15_H_10_O_6_	1.633	151.00/175.04/199.04/217.05/241.05/243.03/257.05	luteolin
**26**	16.44	287.0556	C_15_H_12_O_6_	2.249	151.00	eriodictyol
**27**	16.68	329.0661	C_17_H_14_O_7_	1.67	286.12/314.04/330.18	3′,4′-dimethoxyquercetin
**28**	16.92	373.0919	C_19_H_18_O_8_	0.49	327.14	methylrosmarinic acid
**29**	17.28	207.0658	C_11_H_12_O_4_	3.1	179.03	caffeic acid ethyl ester
**30**	17.99	359.0766	C_18_H_16_O_8_	1.521	344.05/339.98	2-(3,4-dimethoxyphenyl)-5,7,8-trihydroxy-6-methoxy-4H-1-benzopyran-4-one
**31**	18.23	269.0446	C_15_H_10_O_5_	0.632	255.06/149.02/201.06/227.03/183.04	apigenin
**32**	18.47	271.0604	C_15_H_12_O_5_	1.328	151.00/177.02	naringenin
**33**	22.29	165.0918	C_10_H_14_O_2_	4.869	150.07/138.03	thanol
**34**	23.07	285.0757	C_16_H_14_O_5_	−0.035	151.00/175.04/199.04/217.05/241.05/243.03/257.05	7-*O*-methoxy naringenin
**35**	24.31	329.1754	C_20_H_26_O_4_	2.079	286.12/330.18	3,4,3′,4′-tetrahydroxy-5,5′-diisopropyl-2,2′-dimethylbiphenyl
**36**	25.48	313.1802	C_20_H_26_O_3_	1.433	283.13/297.15/269.12/313.18	3,4,4′-trihydroxy-5,5′-diisopropyl-2,2′-dimethylbiphenyl
**37**	26.44	343.1541	C_20_H_24_O_5_	0.320	325.14/315.16/300.10	6,3′,4′-trihydroxy-5,5′-diisopropyl-2,2′-dimethylbiphenyl-3,4-dione
**38**	26.92	327.1593	C_20_H_24_O_4_	−0.838	284.10/299.16/312.14/291.97/269.08	3′,4′-dihydroxy-5,5′-diisopropyl-2,2′-dimethylbiphenyl-3,4-dione
**39**	37.38	455.3511	C_30_H_48_O_3_	1.761	411.33/409.34/343.29	oleanolic acid

**Table 2:** ^13^C and ^1^H NMR (*J* in Hz) of compound **37** (600 MHz, δ ppm, in CD_3_OD).

37		
	δ_C_	δ_H_ (*J* in Hz)
1	146.0	-
2	137.0	-
3	185.0	-
4	186.7	-
5	124.0	-
6	147.0	-
7	12.5	1.80, s
8	25.0	3.25, m
9	19.9	1.26, d (7.20)
10	19.9	1.26, d (7.20)
1′	126.0	-
2′	120.8	-
3′	142.0	-
4′	142.0	-
5′	132.5	-
6′	118.3	6.37, s
7′	13.2	1.91, s
8′	27.6	3.29, m
9′	22.7	1.22, d (7.20)
10′	22.7	1.22, d (7.20)

**Table 3:** Antioxidant activity and flavonoid content of *n*-BuOH extract of *S. bachtiarica* evaluated via spectrophotometric assays.

Material	Total Flavonoids [mg/g Plant Extract (in RE) ± SD ^a^] ^b^	DPPH^•^ IC_50_ ^c^ ± SD
*n*-BuOH extract	162.33 ± 0.43	85.31 ± 1.84
Vitamin C ^d^	-	1.35 ± 0.21

^a^ Standard deviation of two independent experiments. ^b^ Flavonoid content evaluated using aluminium chloride assay and expressed as mg of rutin equivalents (RE) per gram of extract. ^c^ Antioxidant activity determined via DPPH assay. Range of tested concentrations (50–100–200 μg/emL). ^d^ Positive control for DPPH assay.

**Table 4:** Inhibitory activity (expressed in terms of percentage) of *Satureja bachtiarica* extract and purified compounds on the bacterial mature biofilm.

Compound	*A. baumannii*	*E. coli*	*L. monocytogenes*	*P. aeruginosa*	*S. aureus*
**1_40**	0 (0)	28.80 (1.13) ^b^	24.31 (1.17) ^a^	0 (0)	0 (0)
**1_80**	13.09 (1.14) ^a^	54.46 (2.73) ^b^	61.68 (3.69) ^c^	20.34 (3.05) ^a^	26.73 (3.92) ^b^
**2_40**	45.71 (1.65) ^b^	0 (0)	11.32 (1.88) ^a^	22.05 (1.34) ^a^	33.41 (1.22) ^b^
**2_80**	58.71 (1.44) ^c^	38.47 (1.17) ^b^	41.82 (1.64) ^b^	34.86 (3.37) ^b^	52.85 (1.13) ^b^
**4_40**	6.43 (0.63) ^a^	16.36 (2.60) ^a^	28.51 (1.59) ^b^	21.59 (1.29) ^a^	16.13 (1.72) ^a^
**4_80**	11.07 (0.12) ^a^	78.49 (1.03) ^c^	37.02 (2.05) ^b^	31.61 (2.68) ^b^	19.49 (2.18) ^a^
**5_40**	3.60 (0.52)	77.59 (1.11) ^c^	50.41 (1.90) ^b^	24.61(1.90) ^a^	13.81 (1.25) ^a^
**5_80**	17.26 (1.73) ^a^	79.29 (1.08) ^c^	54,99 (2.50) ^b^	32.51(3.37) ^b^	29.44 (1.41) ^b^
**16_40**	0 (0)	9.83 (3.20) ^a^	0 (0)	0 (0)	0 (0)
**16_80**	0 (0)	19.09 (0.97) ^a^	3.70	4.66 (0.54)	6.74 (0.18)
**19_40**	0 (0)	16.39 (0.45) ^a^	0 (0)	0 (0)	0 (0)
**19_80**	0 (0)	29.94 ^b^	0 (0)	20.27 (2.28) ^a^	0 (0)
**21_40**	0 (0)	15.67 (3.28) ^a^	53.69 (0.94) ^b^	45.48 (1.09) ^b^	30.54 (0.93) ^b^
**21_80**	59.04 (0.60) ^c^	50.20 (1.72) ^b^	57.39 (0.99) ^c^	51.65 (0.81) ^b^	45.44 (1.13) ^b^
**23_40**	0 (0)	6.15 (1.25) ^b^	17.52 (1.70) ^a^	3.12 (011)	20.69 (2.80) ^a^
**23_80**	39.84 (4.73) ^b^	30.33 (3.56) ^b^	19.09(1.30) ^a^	29.94 (0.21) ^b^	48.65 (3.85) ^b^
**26_40**	1.40 (1.70)	6.51 (2.42) ^a^	0 (0)	3.62 (0.24)	12.90 (2.19) ^a^
**26_80**	24.45 (2.10) ^b^	20.24 (2.37) ^a^	14.17 (1.84) ^a^	9.57 (0.17) ^a^	44.07 (3.62) ^b^
**27_40**	0 (0)	11.33 (2.50) ^a^	0 (0)	0 (0)	16.96 (2.00) ^a^
**27_80**	0 (0)	12.51 (1.28) ^a^	0 (0)	20.97 (2.51) ^a^	23.35 (2.22) ^a^
**28_40**	0 (0)	0 (0)	4.95 (0.18)	13.06 (1.09) ^a^	9.12 (0.40) ^a^
**28_80**	0 (0)	5.17 (0.31) ^a^	12.64 (1.12) ^a^	27.05 (1.41) ^b^	27.31 (1.90) ^b^
**32_40**	20.80 (1.21) ^a^	31.28 (0.82) ^b^	27.90 (2.11) ^b^	35.05 (1.22) ^b^	51.44 (0.65) ^b^
**32_80**	47.18 (1.23) ^b^	45.93 (0.12) ^b^	47.68 (1.46) ^b^	48.87 (2.26) ^b^	75.49 (2.49) ^c^
**33_40**	7.25 (0.50) ^a^	56.07 (1.33) ^c^	19.52 (0.63) ^a^	17.58 (1.77) ^a^	8.17 (1.35) ^a^
**33_80**	18.59 (2.12) ^a^	60.96 (1.08) ^c^	50.47 (1.99) ^b^	30.81 (2.98) ^b^	26.44 (1.35) ^b^
**35_40**	48.16 (1.33) ^b^	53.01 (1.21) ^b^	60.23 (0.63) ^c^	51.61 (1.12) ^b^	37.10 (1.03) ^b^
**35_80**	52.76 (2.06) ^c^	60.26 (1.34) ^c^	62.13 (2.04) ^c^	68.94 (1.06) ^c^	46.78 (0.51) ^b^
**36_40**	20.80 (1.81) ^a^	43.86 (1.38) ^b^	64.50 (0.85) ^c^	45.80 (1.24) ^b^	45.48 (1.83) ^b^
**36_80**	61.27 (1.57) ^c^	69.28 (1.29) ^c^	75.02 (0.74) ^c^	81.08 (0.89) ^d^	82.94 (1.14) ^d^
**37_40**	53.20 (1.35) ^b^	45.66 (1.22) ^b^	61.17 (0.26) ^c^	54.31 (1.25) ^c^	44.62 (1.01) ^c^
**37_80**	68.53 (1.24) ^c^	45.16 (1.65) ^b^	68.55 (0.81) ^c^	59.38 (1.33) ^c^	53.25 (1.12) ^c^
**38_40**	49.07 (1.18) ^b^	49.84 (1.12) ^b^	65.09 (0.26) ^c^	59.11 (1.17) ^c^	60.62 (0.99) ^c^
**38_80**	74.21 (0.9) ^c^	53.04 (1.65) ^b^	72.36 (0.81) ^c^	64.54 (1.70) ^c^	63.04 (0.68) ^c^
**EXT_40**	18.98 (0.77) ^a^	19.74 (0.58) ^a^	30.26 (2.01) ^b^	20.96 (1.69) ^a^	0 (0)
**EXT_80**	47.99 (1.10) ^b^	40.69 (2.45) ^b^	64.33 (4.57) ^c^	22.49 (1.63) ^a^	0 (0)

The inhibitory activity of samples was tested at 40 (indicated in the table as **_40**) and 80 (indicated in the table as **_80**) μg/mL and was calculated with respect to the control (cells grown without the presence of the samples, for whom the inhibition rate was set at 0%). Results are reported as the mean ± SD of three experiments. ^a^: *p* < 0.05, ^b^: *p* < 0.01, ^c^: *p* < 0.001, ^d^: *p* < 0.0001 according to two-way ANOVA.

**Table 5:** Inhibitory activity (expressed in terms of percentage) of *Satureja bachtiarica* extract and its compounds on the bacterial sessile cell metabolism.

Compound	*A. baumannii*	*E. coli*	*L. monocytogenes*	*P. aeruginosa*	*S. aureus*
**1_40**	11.2 (0.31) ^a^	1.12 (0.025)	43.18 (6.99) ^b^	0 (0)	29,21(2.51) ^b^
**1_80**	52.54 (1.37) ^c^	23.63 (2.18) ^a^	64.80 (2.72) ^c^	0 (0)	55.41 (1.78) ^c^
**2_40**	53.36 (2.83) ^c^	0 (0)	8.36 (2.22) ^a^	8.61 (0.01) ^a^	0 (0)
**2_80**	77.03 (2.77) ^c^	18.26 (1.89) ^a^	40.62 (3.74) ^b^	23.11 (0.49) ^a^	9.97 (1.78) ^a^
**4_40**	62.70 (2.54) ^c^	16.04 (1.31) ^a^	68.81 (2.21) ^c^	0 (0)	44.65 (1.10) ^b^
**4_80**	66.25 (5.80) ^c^	28.55 (2.47) ^b^	70.67 (2.61) ^c^	21.53 (3.80) ^a^	46.49 (0.96) ^b^
**5_40**	54.68 (2.29) ^c^	21.16 (2.22) ^a^	63.48 (1.68) ^c^	0 (0)	50.34 (2.73) ^b^
**5_80**	75.91 (2.08) ^c^	28.14 (2.21) ^b^	64.57 (1.41) ^c^	39.49 (1.19) ^b^	53.47 (2.97) ^b^
**16_40**	44.41 (4.13) ^b^	0 (0)	0 (0)	0 (0)	29.55 (1.43) ^b^
**16_80**	49.92 (3.12) ^b^	15.93 (3.84) ^a^	2.49 (0.19)	23.12 (2.66) ^a^	32.60(1.51) ^b^
**19_40**	50.14 (3.07) ^b^	10.34 (1.75) ^a^	11.21 (0.12) ^a^	0 (0)	0 (0)
**19_80**	55.03 (3.44) ^c^	55.56 (4.42) ^c^	58.64(3.46) ^c^	70.92 (1.78) ^c^	42.99 (3.89) ^b^
**21_40**	53.89 (1.85) ^b^	0 (0)	5.64 (0.43) ^a^	22.11 (2.86) ^a^	9.53 (2.14) ^a^
**21_80**	64.47 (2.39) ^c^	7.97 (1.50) ^a^	32.23 (3.42) ^b^	25.66 (1.89) ^b^	17.71 (2.01) ^a^
**23_40**	55.81 (4.69) ^c^	0 (0)	0 (0)	0 (0)	22.24 (2.37) ^a^
**23_80**	58.06 (4.34) ^c^	0 (0)	22.94 (3.79) ^a^	42.39 (2.27) ^b^	26.88 (1.10) ^b^
**26_40**	26.05 (2.05) ^b^	0 (0)	0 (0)	0 (0)	0 (0)
**26_80**	36.27 (2.56) ^b^	0 (0)	32.55 (3.12) ^b^	12.15 (1.77) ^a^	14.34 (1.23) ^a^
**27_40**	26.48 (2.25) ^b^	29.73 (1.47) ^b^	0 (0)	23.70 (2.37) ^a^	19.95 (2.11) ^a^
**27_80**	59.05 (4.45) ^c^	52.60 81.07) ^b^	0 (0)	35.41 (3.05) ^b^	31.59 (1.28) ^b^
**28_40**	52.74 (2.55) ^b^	33.03 (0.96) ^b^	0 (0)	9.32 (1.98) ^a^	0 (0)
**28_80**	56.59 (3.34) ^c^	57.03(1.73) ^c^	0.49 (0.01)	11.98 (0.30) ^a^	0 (0)
**32_40**	35.62 (2.81) ^b^	62.46 (1.29) ^c^	27.44 (2.30) ^b^	34.52 (2.02) ^b^	14.33 (1.90) ^a^
**32_80**	70.71 (3.33) ^c^	72.93 (2.34) ^c^	38.51(1.55) ^b^	50.37 (1.59) ^b^	36.47 (2.18) ^b^
**33_40**	45.22 (0.87) ^b^	22.01(2.61) ^a^	60.41 (1.56) ^c^	0 (0)	49.90 (2.31) ^b^
**33_80**	53.94 (2.57) ^b^	27.03 (1.56) ^b^	67.49 (1.99) ^c^	0 (0)	59.18 (2.13) ^c^
**35_40**	32.22 (2.99) ^b^	0 (0)	0 (0)	27.80 (0.42) ^b^	13.39 (0.42) ^a^
**35_80**	59.65 (1.41) ^c^	37.11 (2.37) ^b^	1.56 (0.02)	38.99 (3.05) ^b^	48.49 (2.12) ^b^
**36_40**	34.78 (2.90) ^b^	43.14 (1.64) ^b^	30.26 (2.41) ^b^	34.72 (4.42) ^b^	39.41 (3.88) ^b^
**36_80**	50.46 (2.55) ^b^	61.83 (2.95) ^c^	53.51 (4.66) ^b^	58.92 (4.34) ^c^	63.68 (2.24) ^c^
**37_40**	33.24 (1.07) ^b^	26.41 (0.77) ^b^	26.42 (1.51) ^c^	24.53 (1.41) ^b^	22.48 (2.01) ^b^
**37_80**	45.27 (0.98) ^b^	48.22 (1.85) ^c^	38.58 (1.85) ^c^	47.22 (1.13) ^c^	52.37 (1.02) ^c^
**38_40**	31.14 (3.05) ^b^	39.11 (0.55) ^b^	56.54 (1.57) ^c^	54.89 (1.91) ^b^	45.68 (3.34) ^b^
**38_80**	47.89 (0.74) ^b^	61.47 (1.85) ^c^	70.69 (2.35) ^c^	57.62 (1.93) ^c^	68.93 (0.91) ^c^
**EXT_40**	61.95 (2.68) ^c^	7.27 (2.22) ^a^	53.28 (2.16) ^b^	0 (0)	33.66 (2.48) ^b^
**EXT_80**	67.35 (2.09) ^c^	28.72 (1.97) ^b^	55.32 (2.65) ^c^	7.86 (0.25) ^a^	47.49 (1.07) ^b^

The inhibitory activity of samples was tested at 40 (indicated in the table as **_40**) and 80 (indicated in the table as **_80**) μg/mL against *A. baumannii*, *E. coli*, *L. monocytogenes*, *P. aeruginosa*, and *S. aureus* and was calculated with respect to the control (cells grown without the presence of the samples, for whom we assumed an inhibition rate = 0%). Results are reported as the mean ± SD of three experiments ^a^ *p* < 0.05, ^b^ *p* < 0.01, ^c^ *p* < 0.001 according to two-way ANOVA.

## Data Availability

Data are contained within the article and Supplementary Materials.

## Supplementary Materials

The following supporting information can be downloaded at: https://www.mdpi.com/article/10.3390/plants13010067/s1, Total Flavonoid Content and DPPH assays; Figures S1–S6: MS and NMR spectra of compound **37**. Figures S7–S36: ^1^H NMR spectra of isolated compounds.
