# Supplementary material for: Phytochemical Investigation and Biofilm-Inhibitory Activity of Bachtiari Savory (Satureja bachtiarica Bunge) Aerial Parts
Source: Plants (Basel). 2023 Dec 25;13(1):67. doi: 10.3390/plants13010067 (PMC10780387; doi:10.3390/plants13010067)
Supplement: Supplementary file 1 [file plants-13-00067-s001.zip › plants-2746510-supplementary.pdf]

Phytochemical investigation and biofilm-inhibitory activity of Bachtiari savory (*Satureja bachtiarica* Bunge) aerial parts

Marzieh Rahmani Samani[1,2], Gilda D'Urso[1], Filomena Nazzaro[3], Florinda Fratianni[3], Milena Masullo[1], Sonia Piacente[1,*]

1 Department of Pharmacy, University of Salerno, Via Giovanni II n. 132, 84084 Fisciano, Italy;
mrahmanisamani@unisa.it (M.R.S.); gidurso@unisa.it (G.D.); mmasullo@unisa.it (M.M.)

2 PhD Program in Drug Discovery and Development, University of Salerno, via Giovanni Paolo II n. 132, 84084 Fisciano, Italy

3 Institute of Food Science-National Research Council (CNR-ISA), Via Roma, 64, 83100 Avellino, Italy;
filomena.nazzaro@isa.cnr.it (F.N.); fratianni@isa.cnr.it (F.F.)

* Correspondence: piacente@unisa.it; Tel.: +39-0899-69763

Total Flavonoid Content

To quantify the total flavonoid content, the allumine chloride colorimetric assay was carried out and rutin was used as a reference. Following that, a 10-milliliter volumetric flask was filled with 1 mL of a sample with a concentration of 1 mg/mL and 4 mL of water. Following this, 0.3 mL of 5% $NaNO_2$ and 0.3 mL of 10% $AlCl_3$ were both added to the flask for 5 minutes. After 6 minutes, 2 mL of 1 M NaOH and the final 10 mL of water were added to the solution. After thoroughly combining the solutions, a UV-visible spectrophotometer was used to detect the absorbance at 510 nm in comparison to a blank control. The amount of flavonoids in the extract was expressed in rutin equivalents (RE), according to equation: $(A_e \times m_0 \times 10)/(A_0 \times m_e)$.

In the equation, A_e represents the average of the absorbance of the extract in triplicate, A_0 represents the average of the absorbance of the standard rutin solution in triplicate, m_e represents the weight of the plant extract that was analyzed in g, and m_0 represents the weight of the rutin in the solution in g. Flavonoid amount was expressed in mg/g plant extract in RE.

DPPH• Radical Scavenging Activity

For DPPH, the percentage of DPPH• radical scavenging activity (%) was plotted against the extract concentration (µg/mL) to determine the $IC_{50.}$ In brief, a stock solution (1 mg/mL) of the extract was used in the range of 50–200ug/mL, and an aliquot (5.0 µL) of the methanol solution containing different amounts of the extract was added to 195 µL of daily prepared DPPH• solution. Absorbance at 517 nm was measured on a UV-visible spectrophotometer (Multiskans skyhigh, Thermo Fisher Scientific, Milan, Italy) 10 min after starting the reaction.

All the experiments were performed in triplicate. Vitamin C provided as a positive control.

List of Figures

Figure S1. ESI/LTQOrbitrap spectrum of compound **37**
Figure S2. MS/MS Spectrum of compound **37**
Figure S3. ^{1}H NMR Spectrum of compound **37**
Figure S4. HSQC Spectrum of compound **37**
Figure S5. HMBC Spectrum of compound **37**
Figure S6. ^{13}C NMR Spectrum of compound **37**.
Figure S7. ^{1}H NMR Spectrum of compound **1**
Figure S8. ^{1}H NMR Spectrum of compound **2**
Figure S9. ^{1}H NMR Spectrum of compound **3**
Figure S10. ^{1}H NMR Spectrum of compound **4**
Figure S11. ^{1}H NMR Spectrum of compound **5**
Figure S12. ^{1}H NMR Spectrum of compound **6**
Figure S13. ^{1}H NMR Spectrum of compound **7**
Figure S14. ^{1}H NMR Spectrum of compound **8**
Figure S15. ^{1}H NMR Spectrum of compound **9**
Figure S16. ^{1}H NMR Spectrum of compound **10**
Figure S17. ^{1}H NMR Spectrum of compound **12**
Figure S18. ^{1}H NMR Spectrum of compound **13**
Figure S19. ^{1}H NMR Spectrum of compound **15**
Figure S20. ^{1}H NMR Spectrum of compound **19**
Figure S21. ^{1}H NMR Spectrum of compound **21**
Figure S22. ^{1}H NMR Spectrum of compound **22**
Figure S23. ^{1}H NMR Spectrum of compound **23**
Figure S24. ^{1}H NMR Spectrum of compound **24**
Figure S25. ^{1}H NMR Spectrum of compound **26**
Figure S26. ^{1}H NMR Spectrum of compound **27**
Figure S27. ^{1}H NMR Spectrum of compound **28**
Figure S28. ^{1}H NMR Spectrum of compound **29**
Figure S29. ^{1}H NMR Spectrum of compound **30**
Figure S30. ^{1}H NMR Spectrum of compound **32**
Figure S31. ^{1}H NMR Spectrum of compound **33**
Figure S32. ^{1}H NMR Spectrum of compound **34**
Figure S33. ^{1}H NMR Spectrum of compound **35**
Figure S34. ^{1}H NMR Spectrum of compound **36**
Figure S35. ^{1}H NMR Spectrum of compound **38**
Figure S36. ^{1}H NMR Spectrum of compound **39**

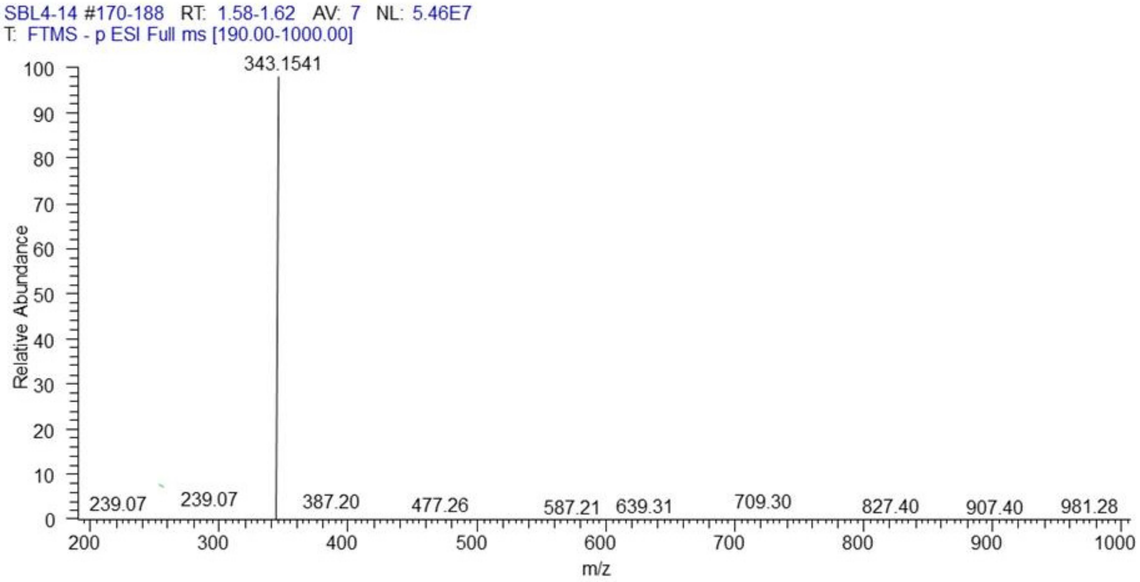

Figure S1. ESI/LTQOrbitrap spectrum of compound **37**, in negative ion mode.

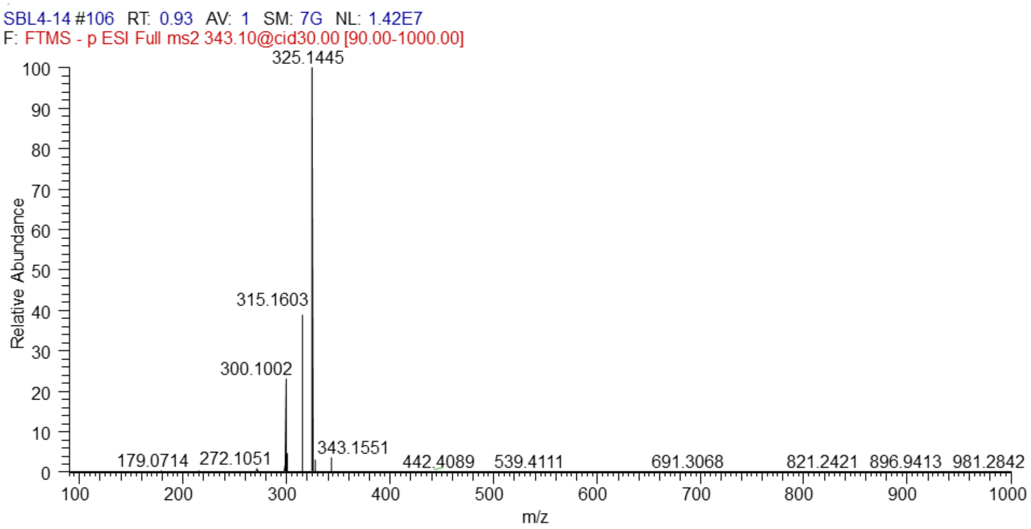

Figure S2. MS/MS spectrum of compound **37**

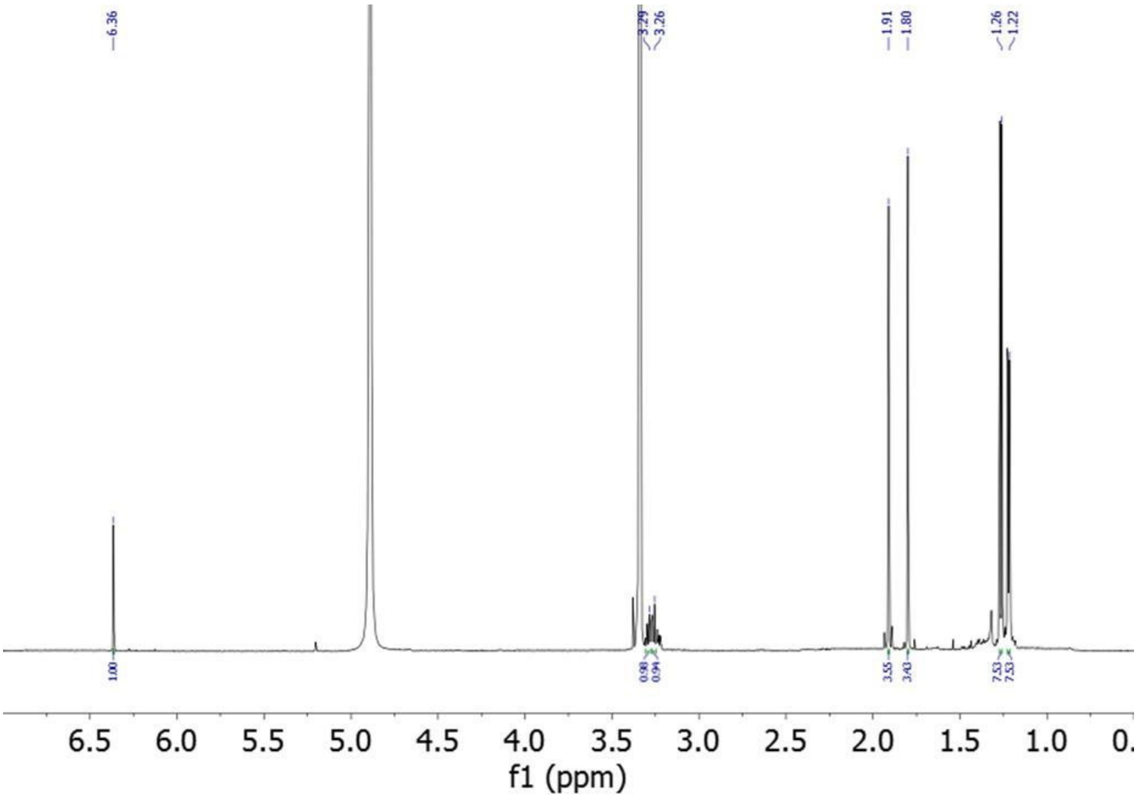

Figure S3. ^{1}H NMR Spectrum (600 MHz, CD_3OD) of compound **37**.

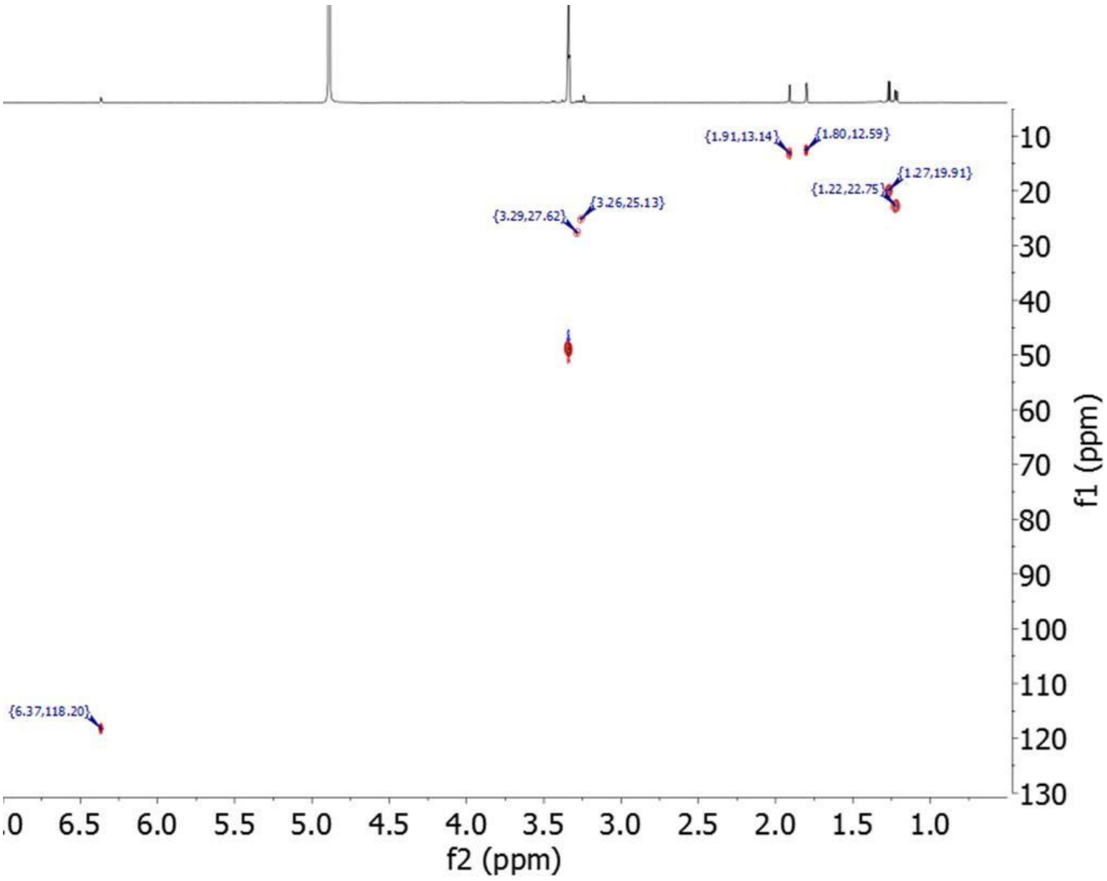

Figure S4. HSQC Spectrum (CD_3OD) of compound **37**.

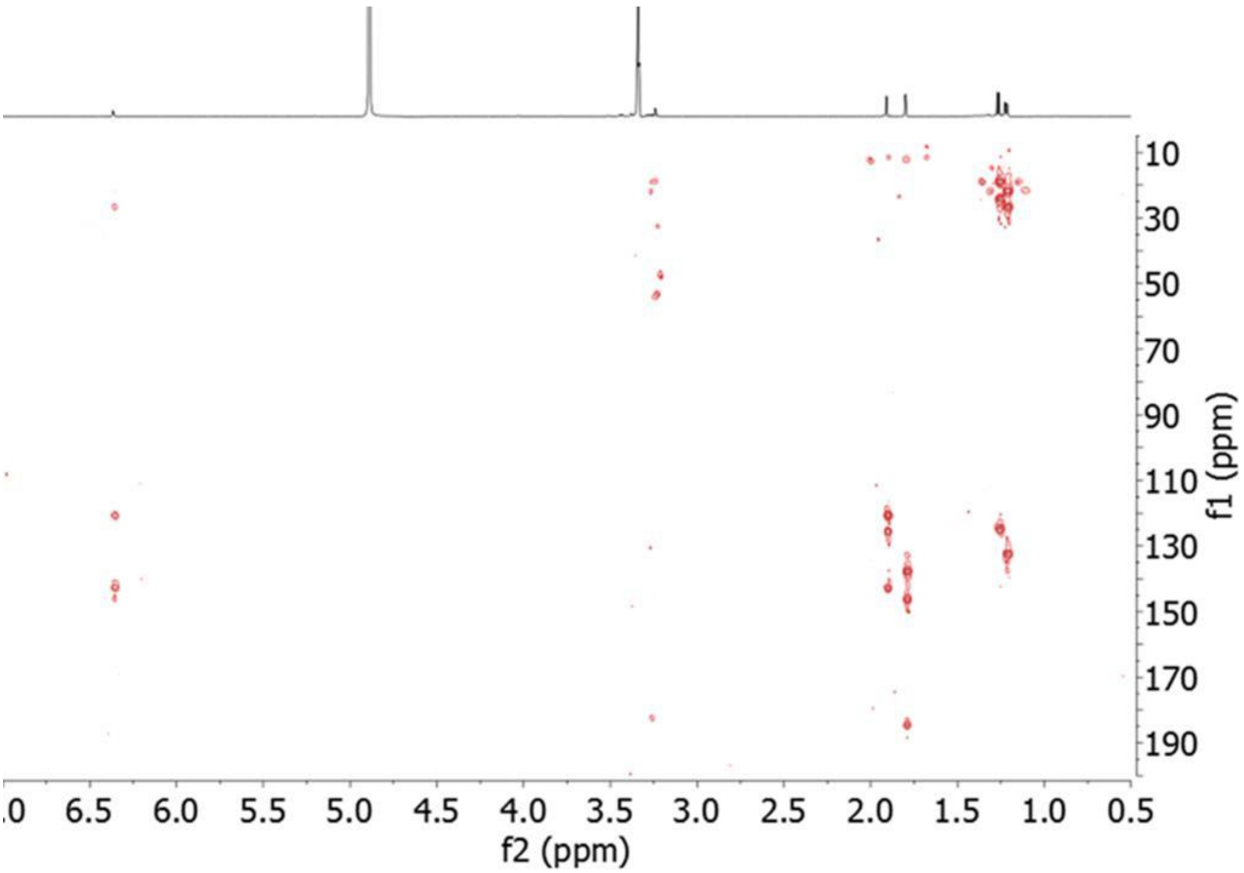

Figure S5. HMBC Spectrum (CD_3OD) of compound **37**.

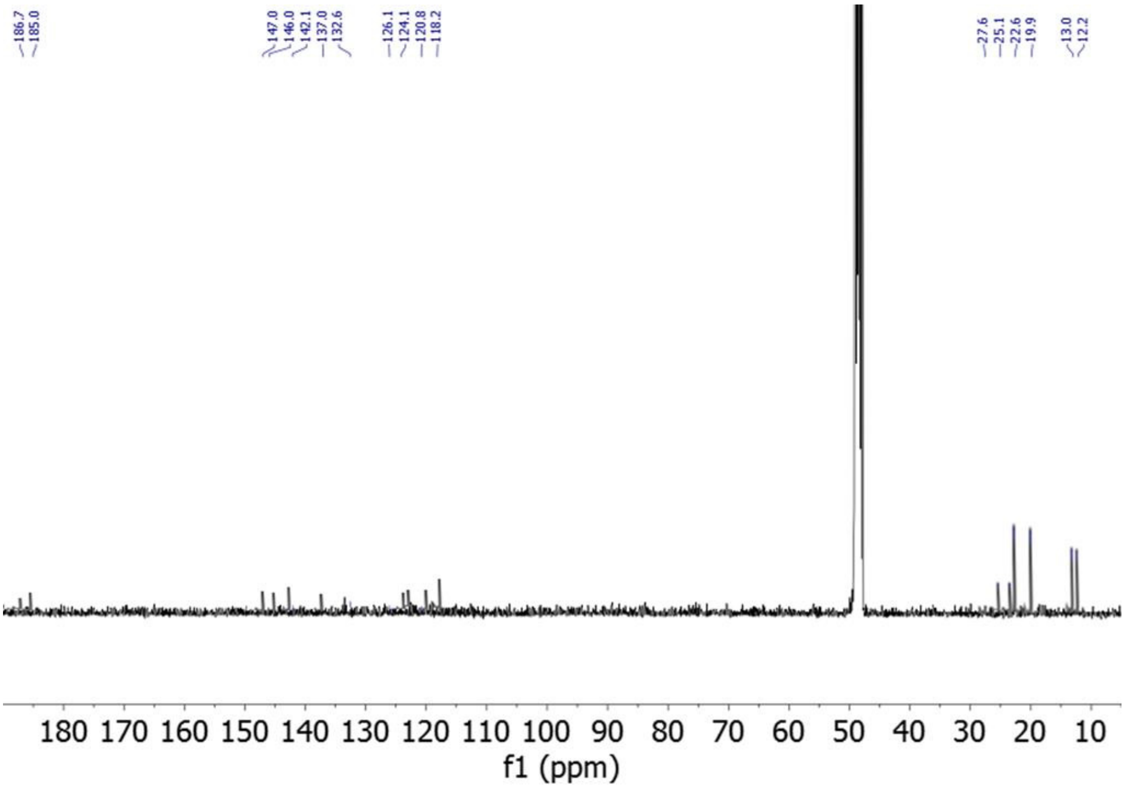

Figure S6. ^{13}C NMR Spectrum (150 MHz, CD_3OD) of compound **37**.

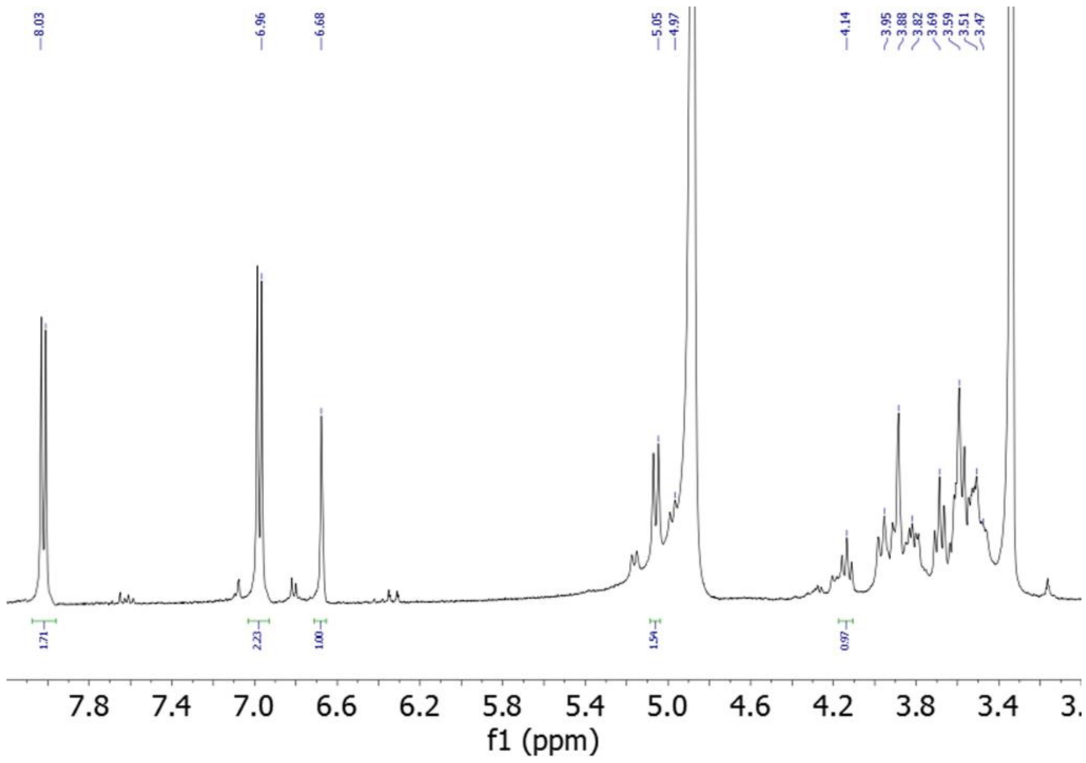

Figure S7. ^{1}H NMR Spectrum (600 MHz, CD_3OD) of compound **1**.

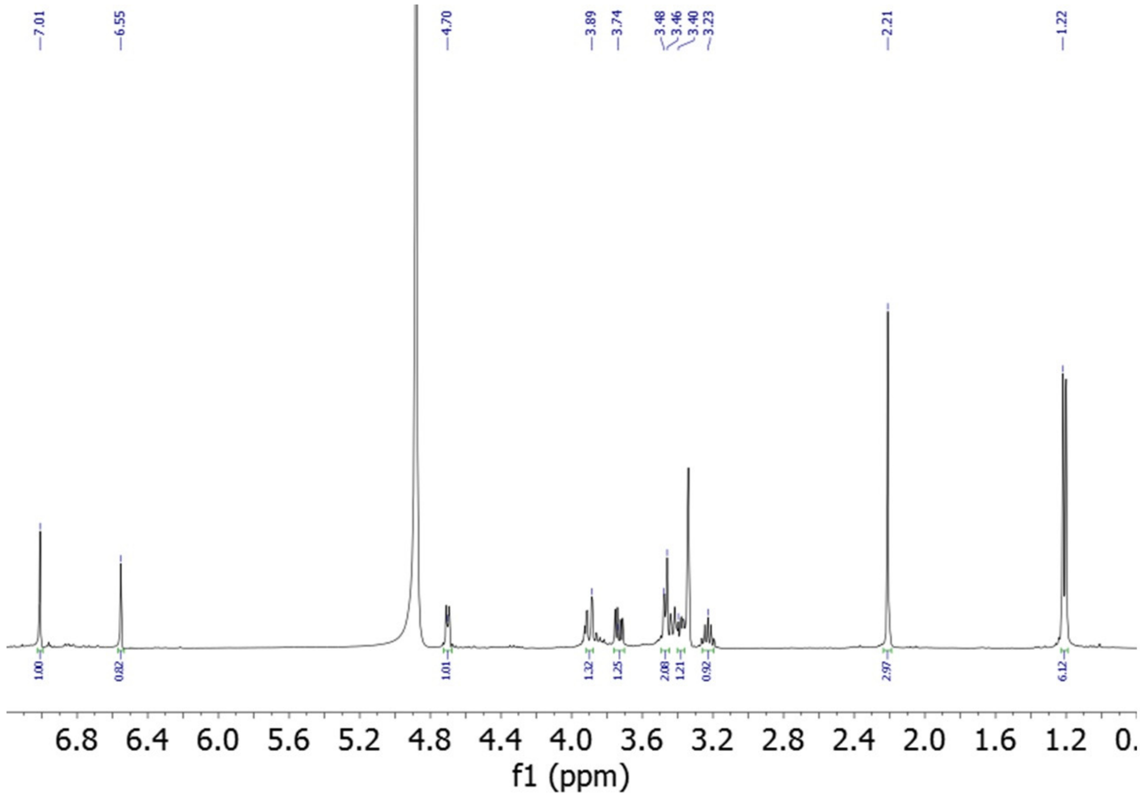

Figure S8. ^{1}H NMR Spectrum (600 MHz, CD_3OD) of compound **2**.

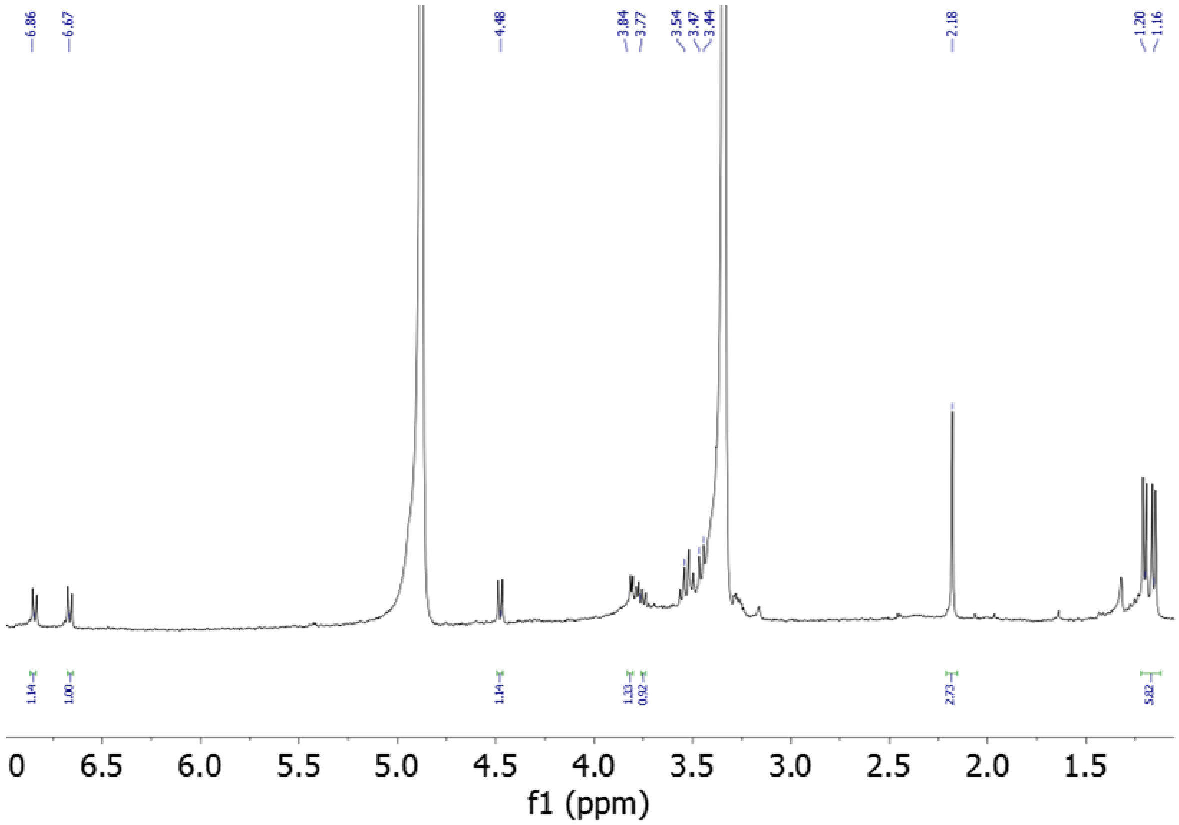

Figure S9. ^{1}H NMR Spectrum (600 MHz, CD_3OD) of compound **3**.

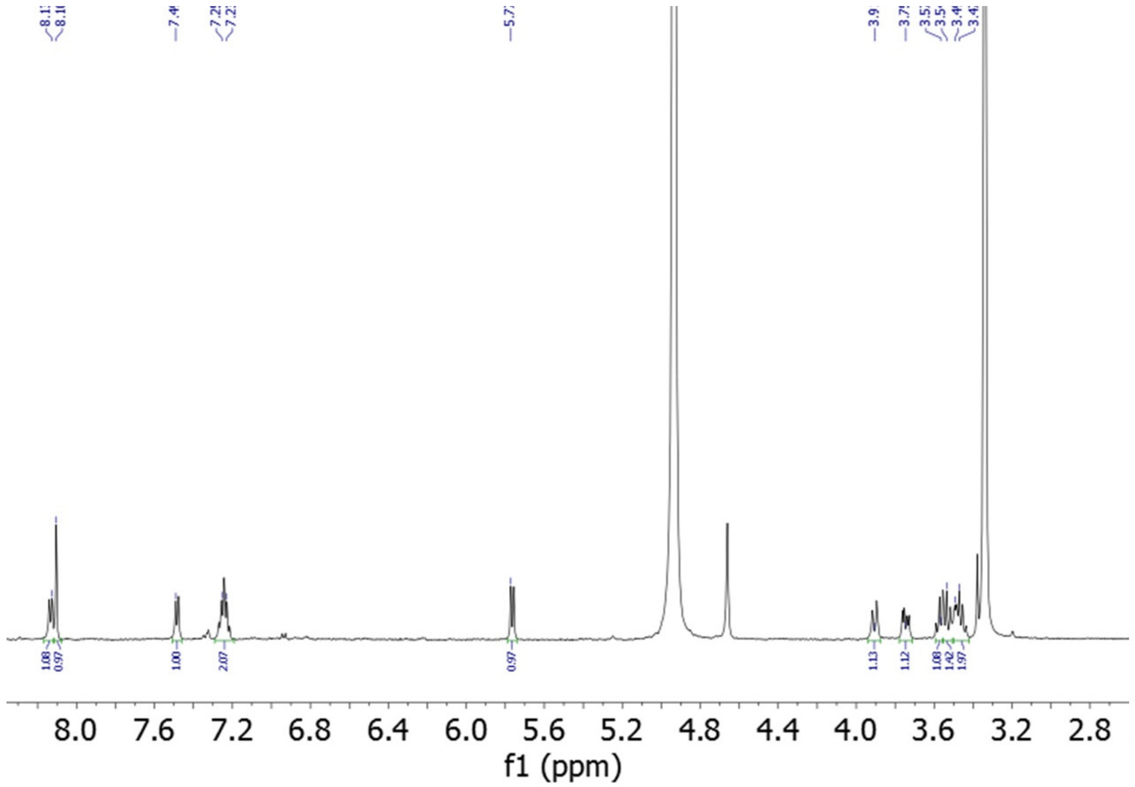

Figure S10. ^{1}H NMR Spectrum (600 MHz, CD_3OD) of compound **4**.

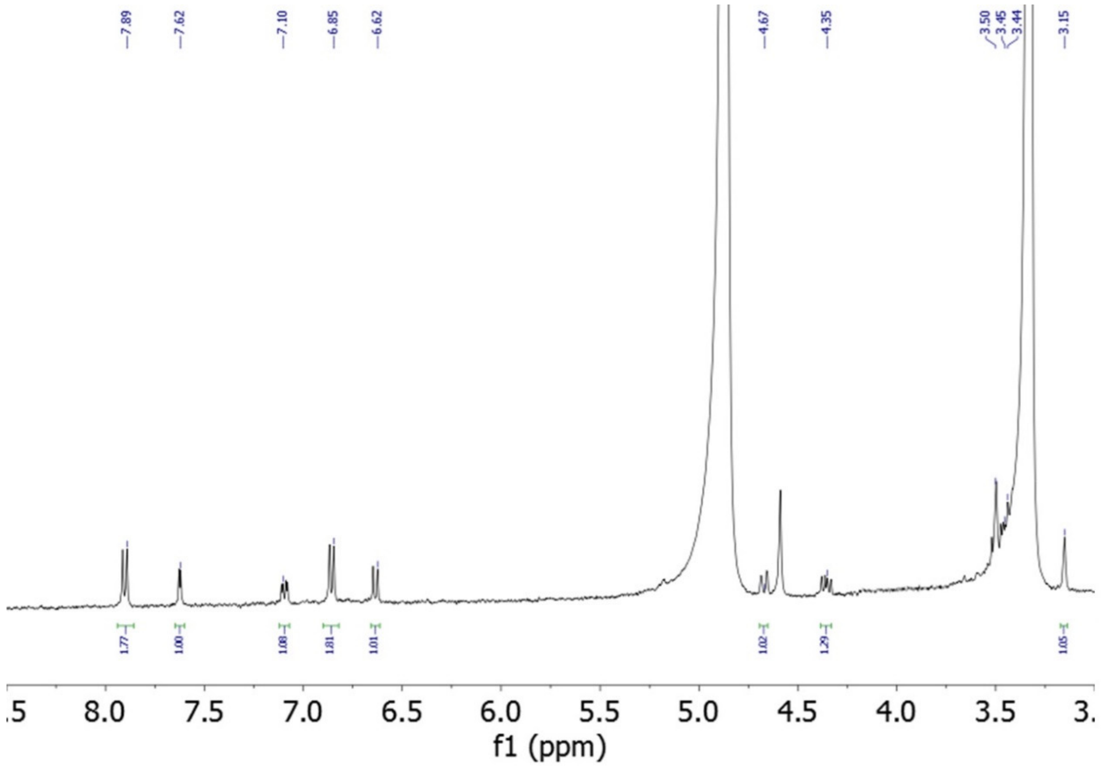

Figure S11. ^{1}H NMR Spectrum (600 MHz, CD_3OD) of compound **5**.

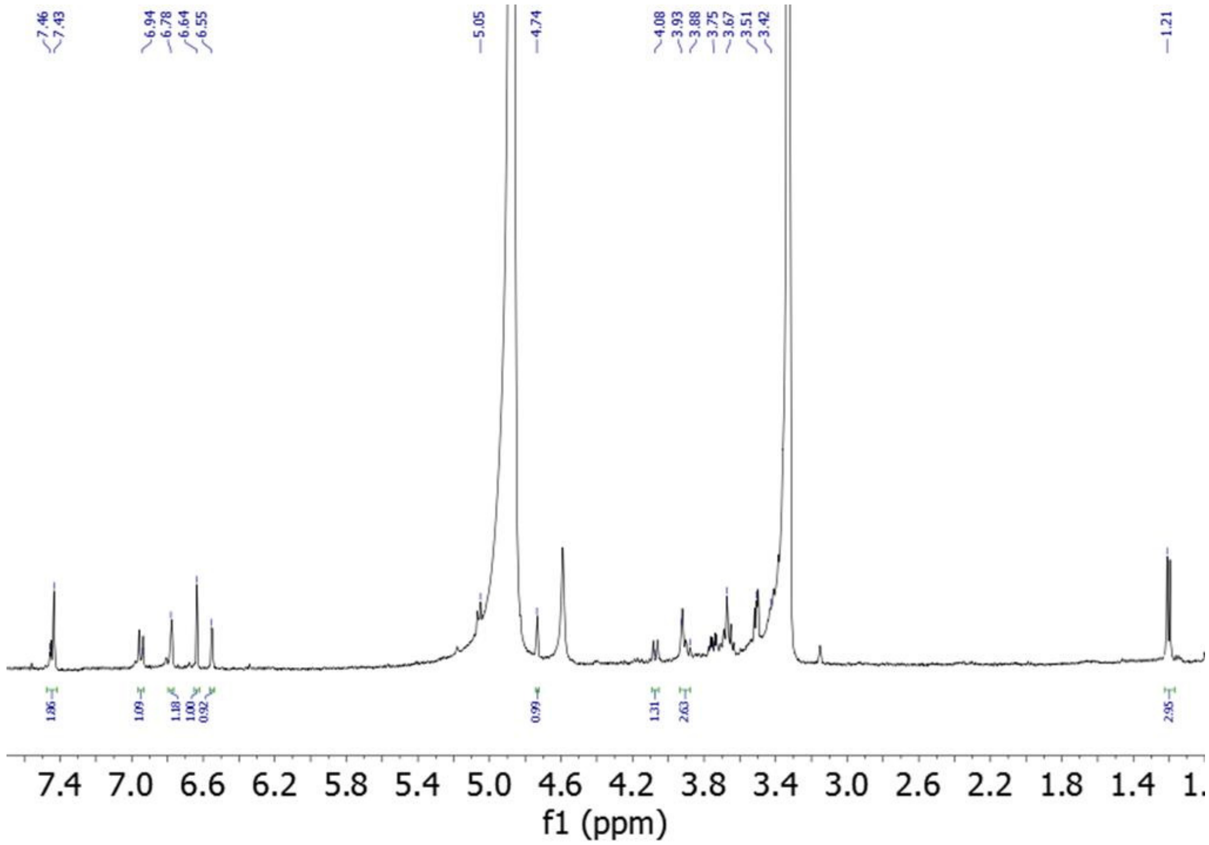

Figure S12. ^{1}H NMR Spectrum (600 MHz, CD_3OD) of compound **6**.

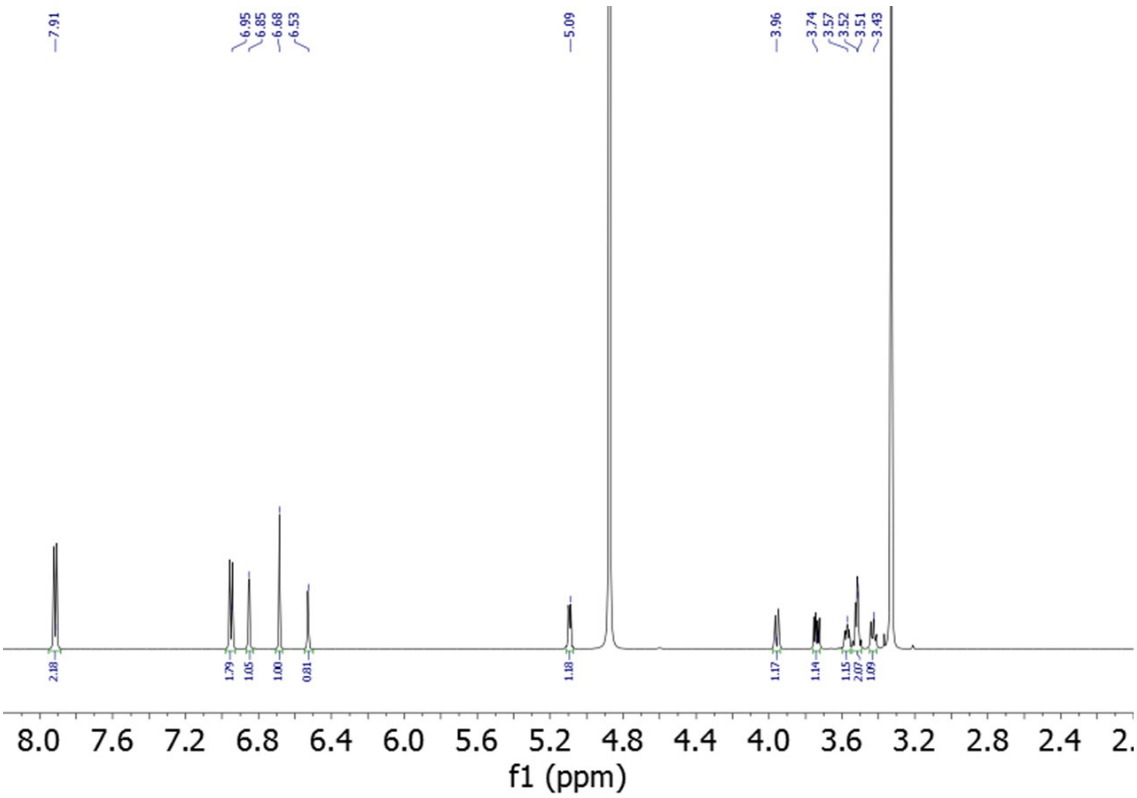

Figure S13. ^{1}H NMR Spectrum (600 MHz, CD_3OD) of compound **7**.

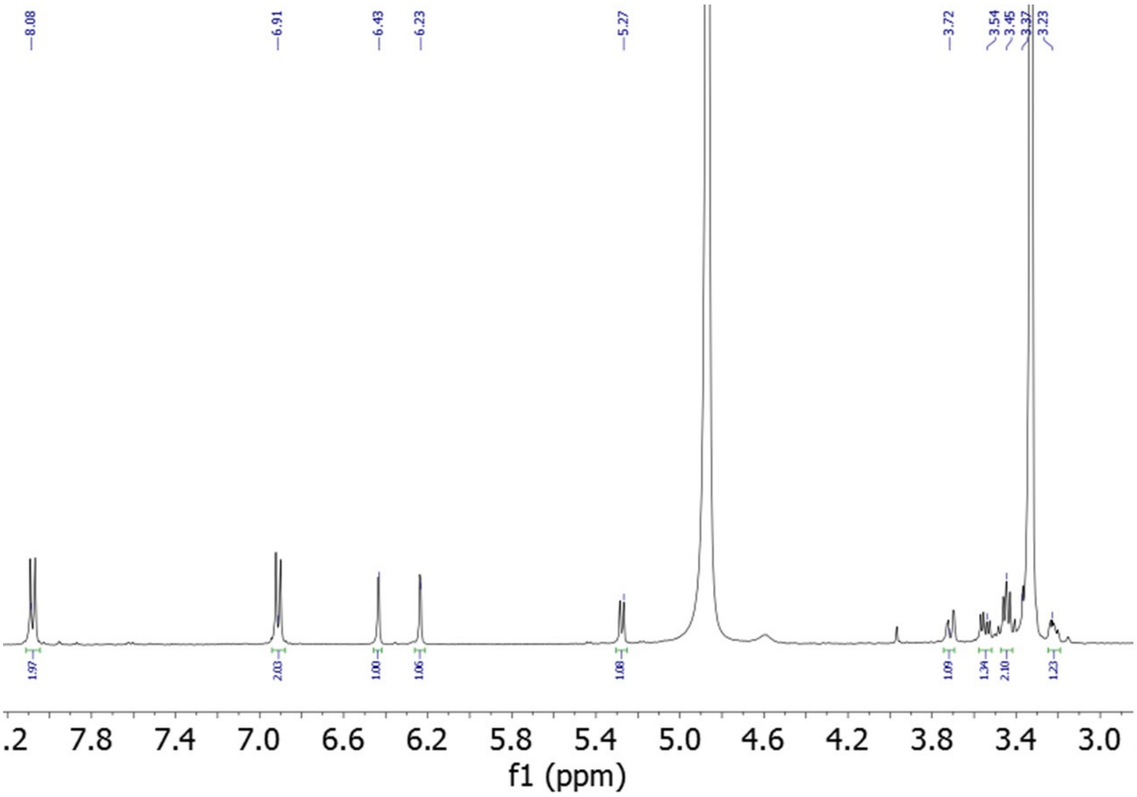

Figure S14. ^{1}H NMR Spectrum (600 MHz, CD_3OD) of compound **8**.

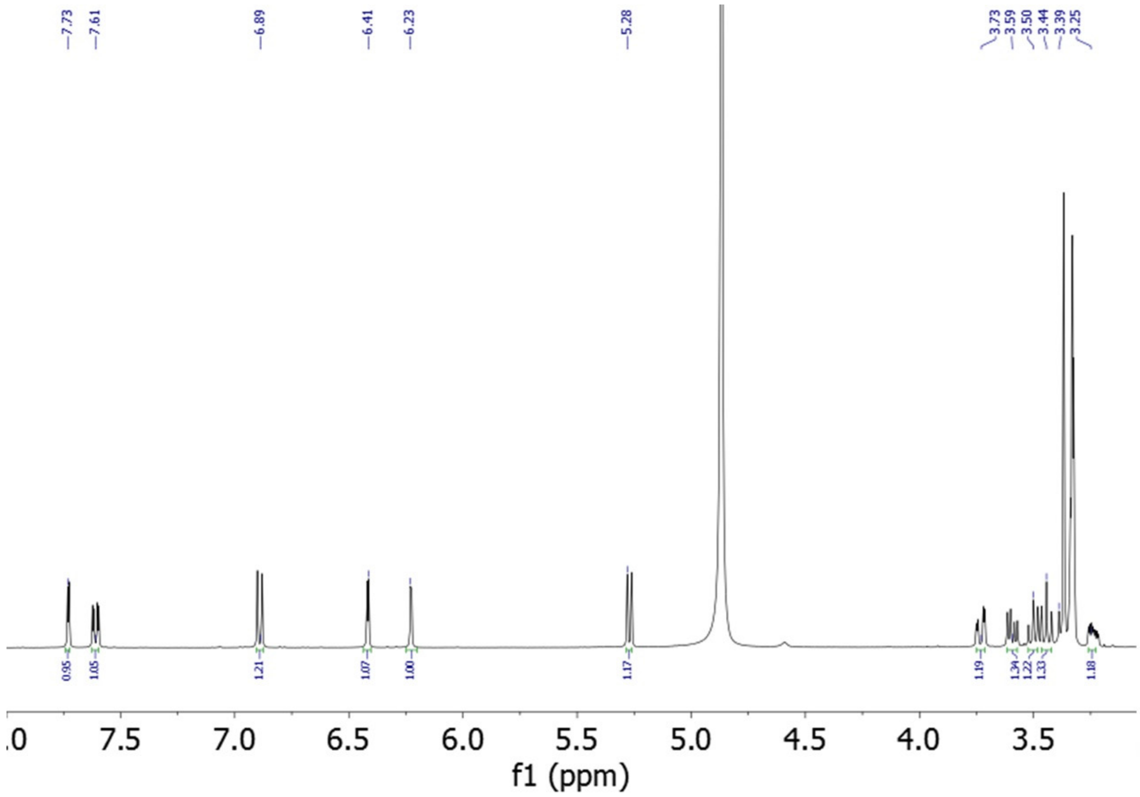

Figure S15. ^{1}H NMR Spectrum (600 MHz, CD_3OD) of compound **9**.

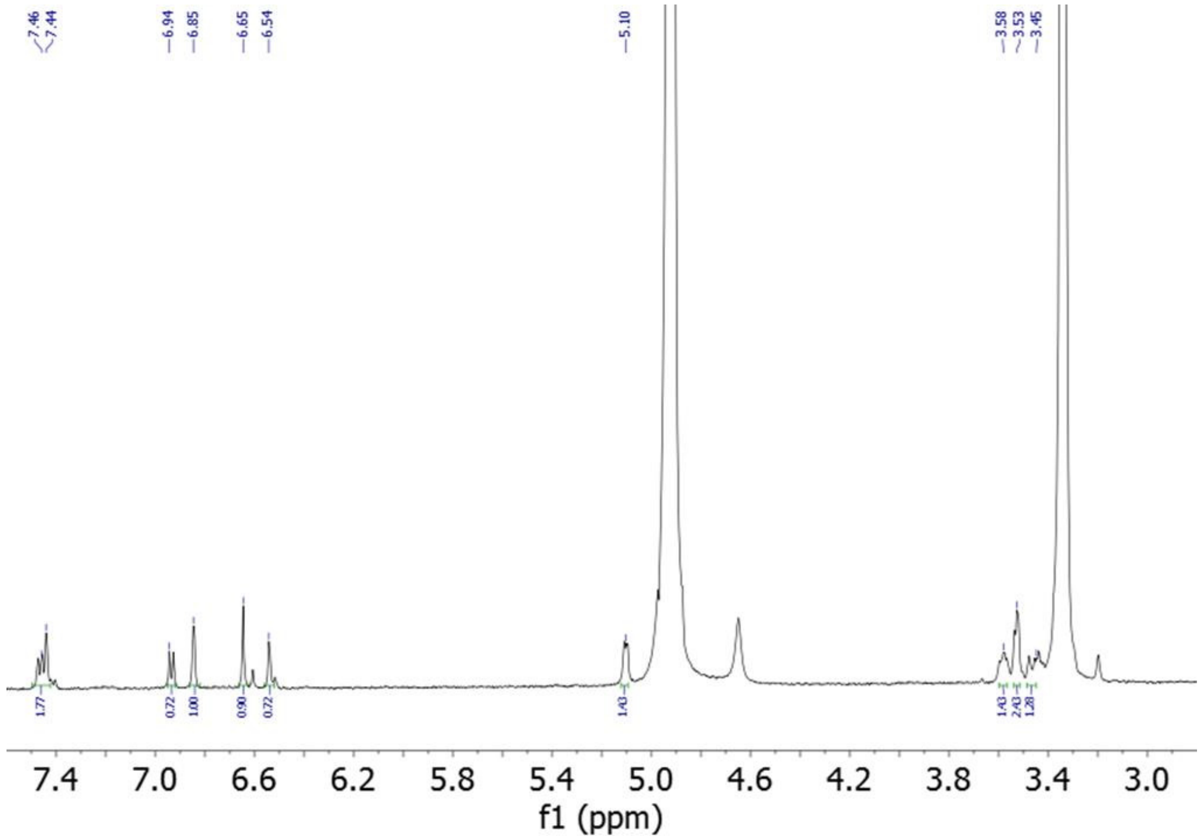

Figure S16. ^{1}H NMR Spectrum (600 MHz, CD_3OD) of compound **10**.

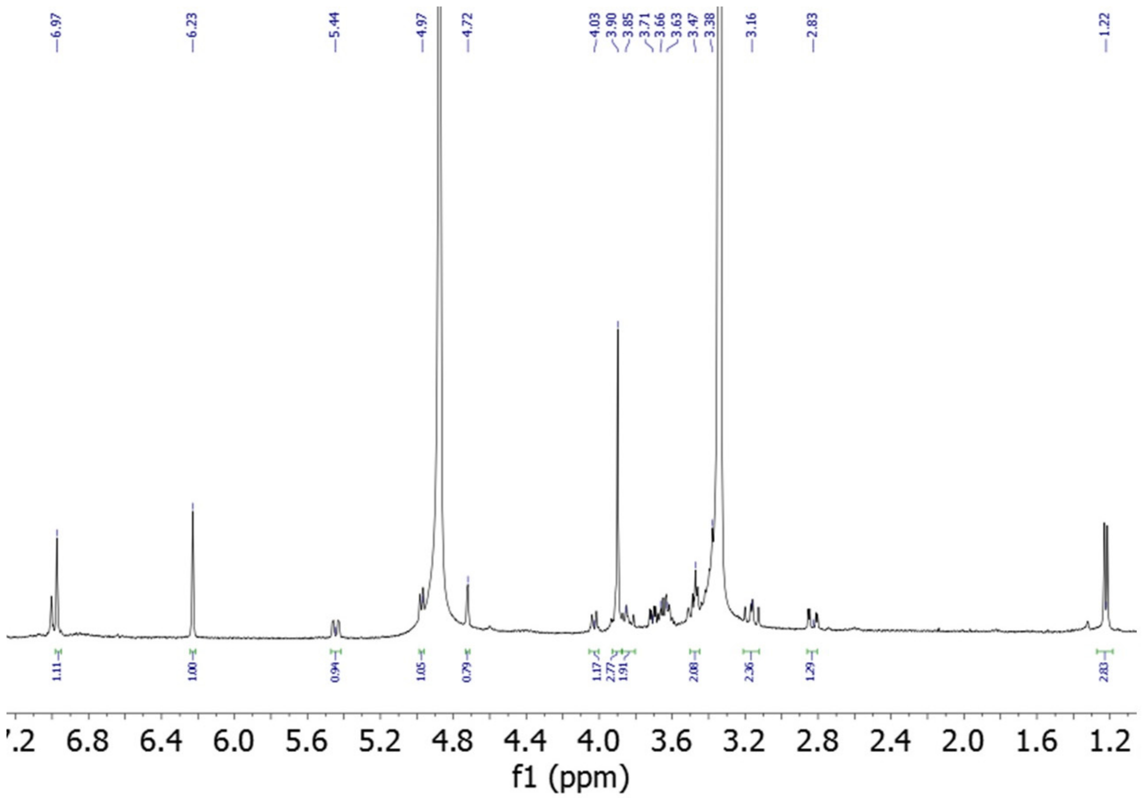

Figure S17. ^{1}H NMR Spectrum (600 MHz, CD_3OD) of compound **12**.

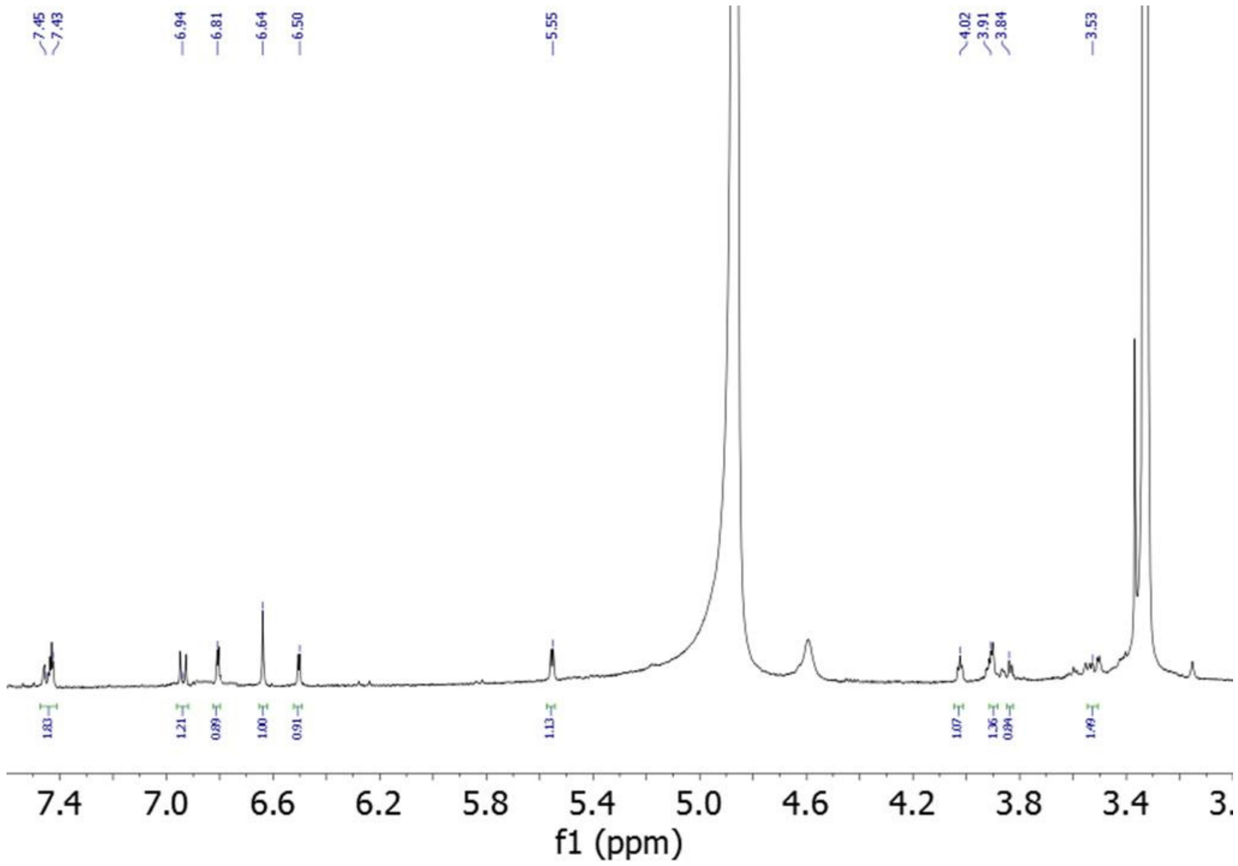

Figure S18. ^{1}H NMR Spectrum (600 MHz, CD_3OD) of compound **13**.

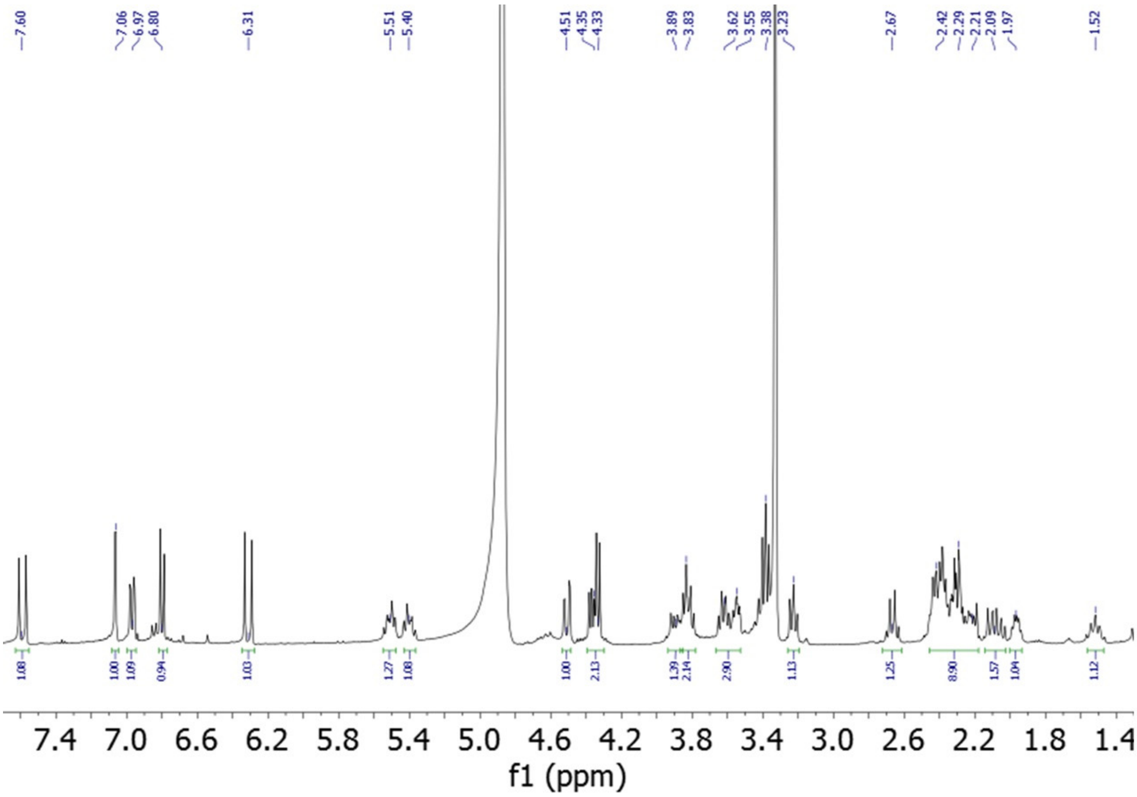

Figure S19. ^{1}H NMR Spectrum (600 MHz, CD_3OD) of compound **15**.

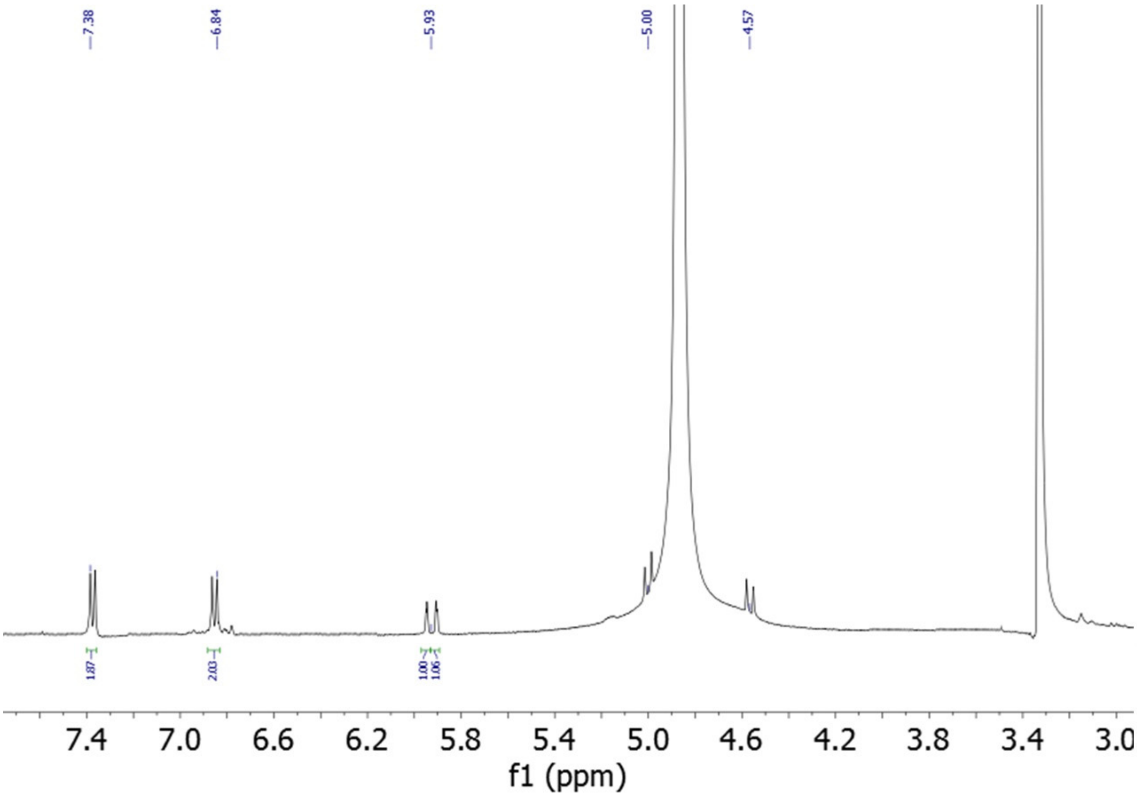

Figure S20. ^{1}H NMR Spectrum (600 MHz, CD_3OD) of compound **19**.

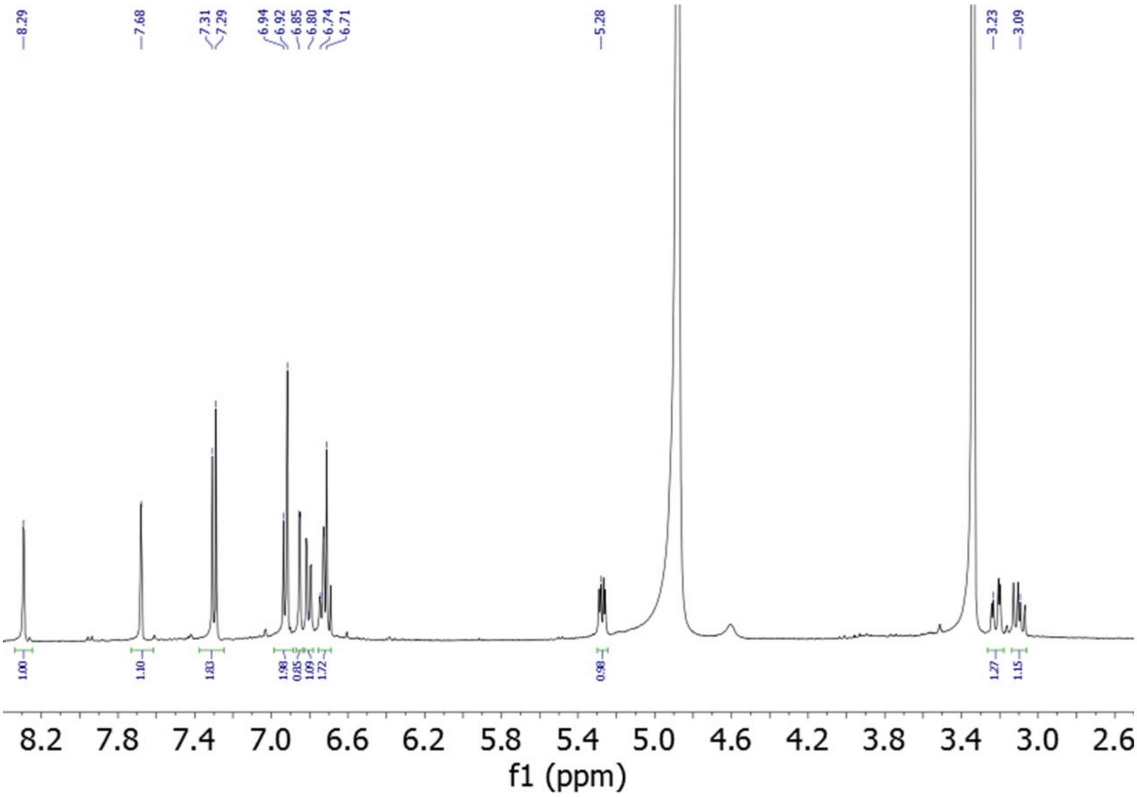

Figure S21. ^{1}H NMR Spectrum (600 MHz, CD_3OD) of compound **21**.

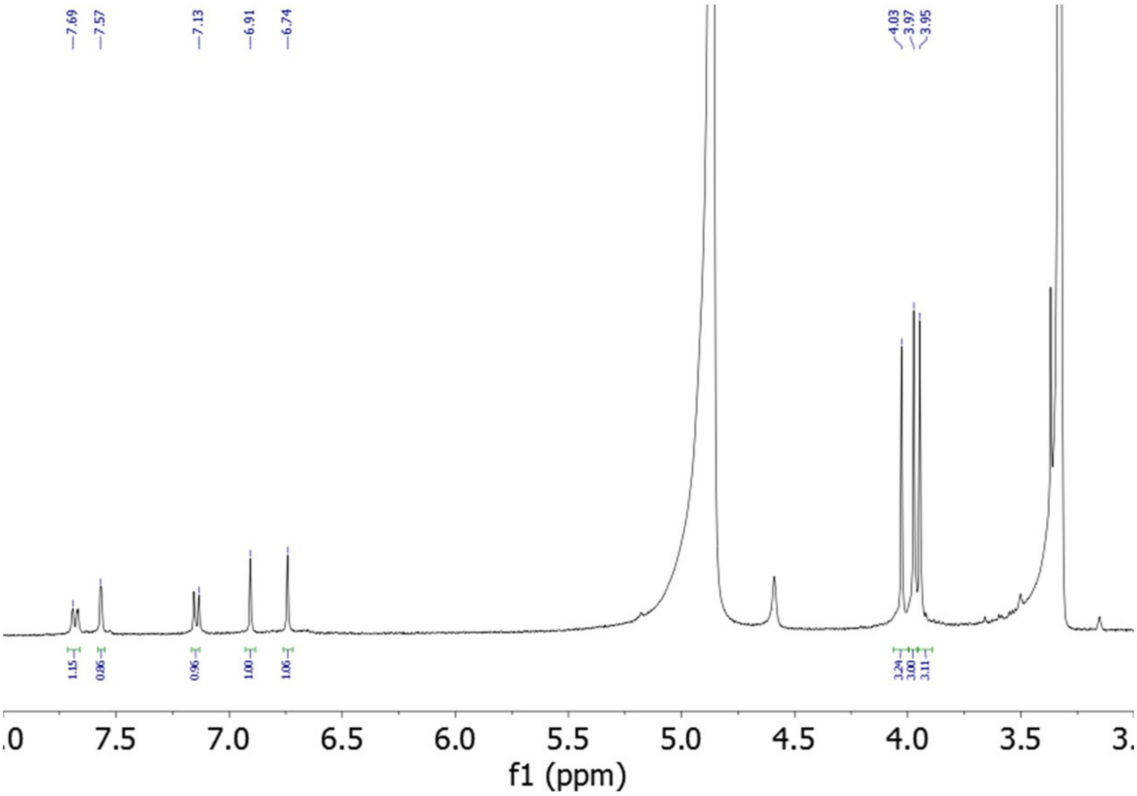

Figure S22. ^{1}H NMR Spectrum (600 MHz, CD_3OD) of compound **22**.

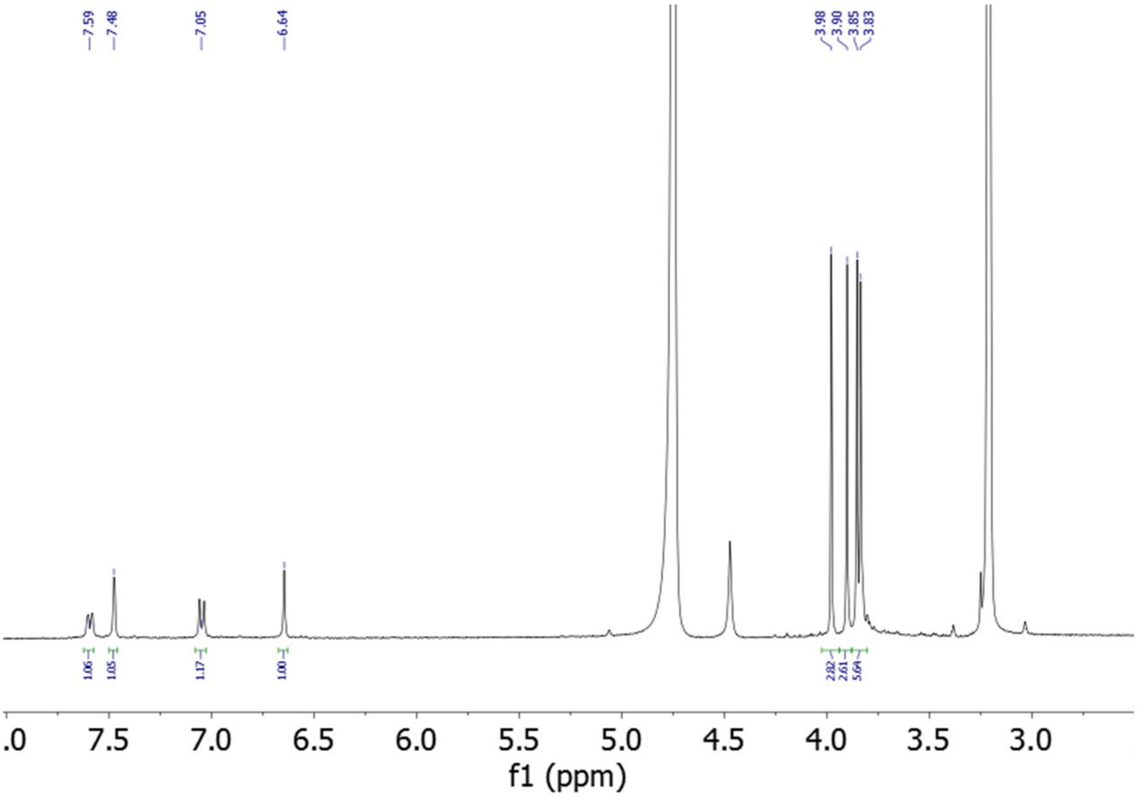

Figure S23. ^{1}H NMR Spectrum (600 MHz, CD_3OD) of compound **23**.

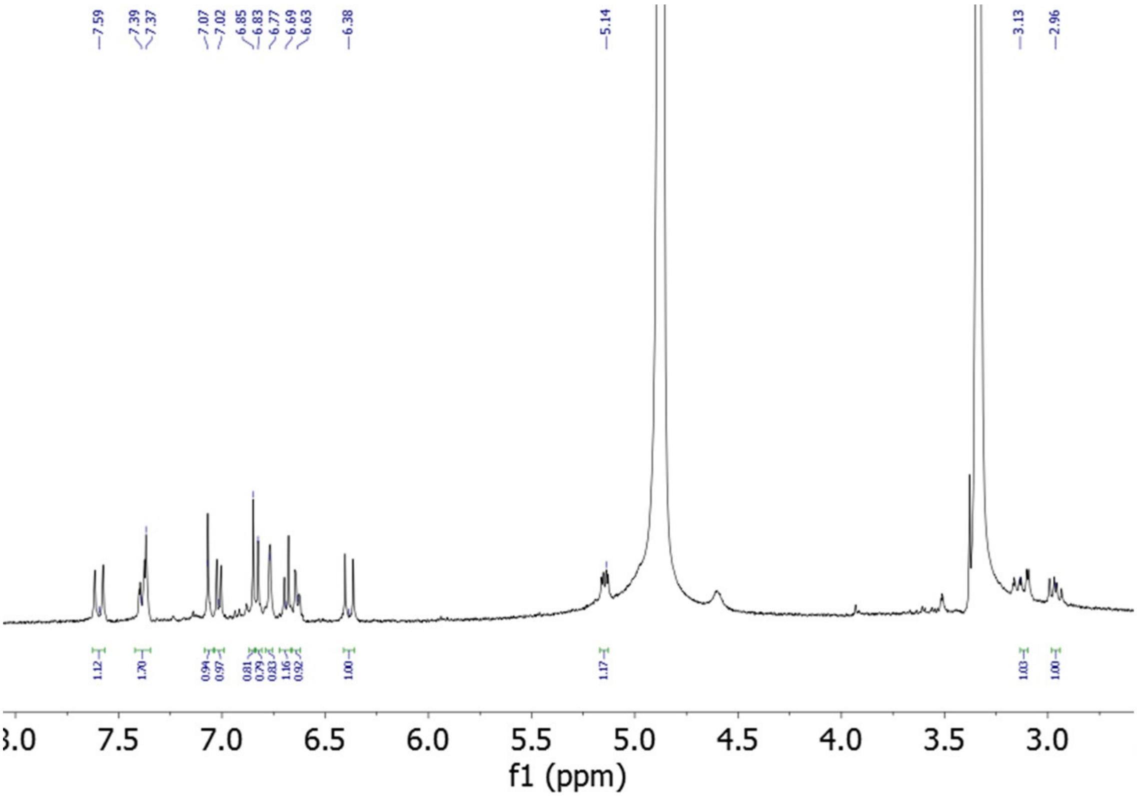

Figure S24. ^{1}H NMR Spectrum (600 MHz, CD_3OD) of compound **24**.

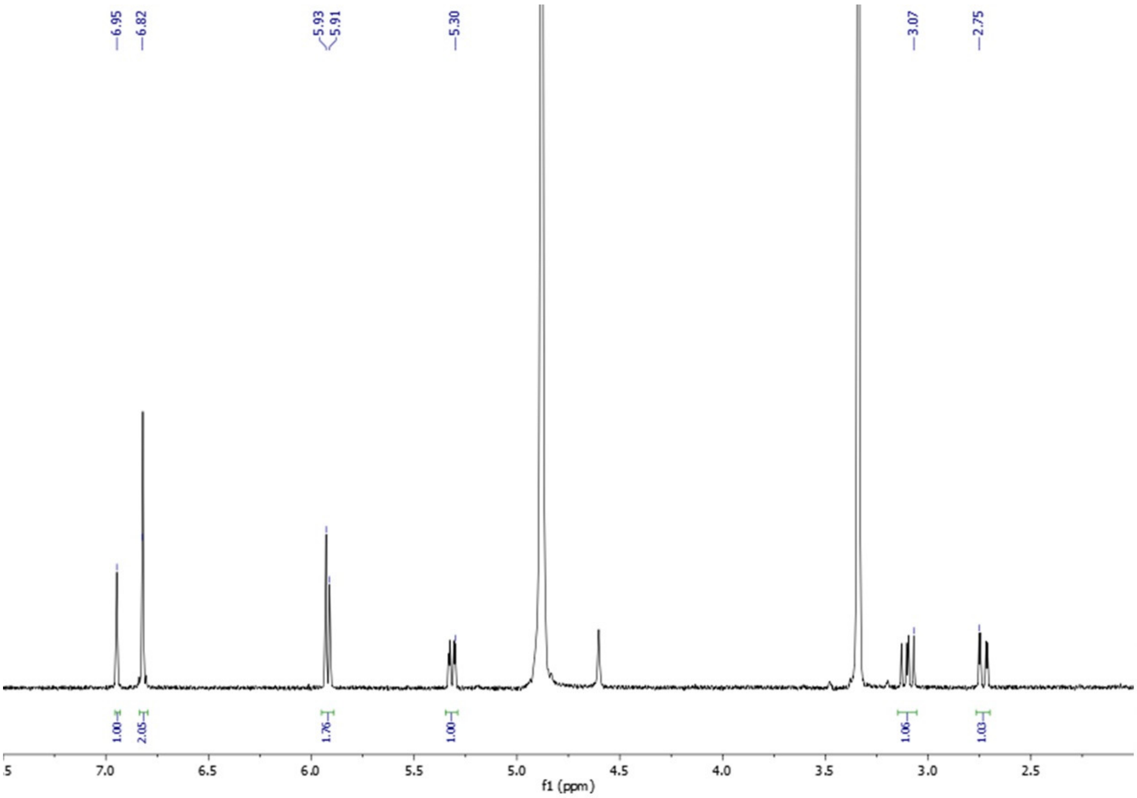

Figure S25. ^{1}H NMR Spectrum (600 MHz, CD_3OD) of compound **26**.

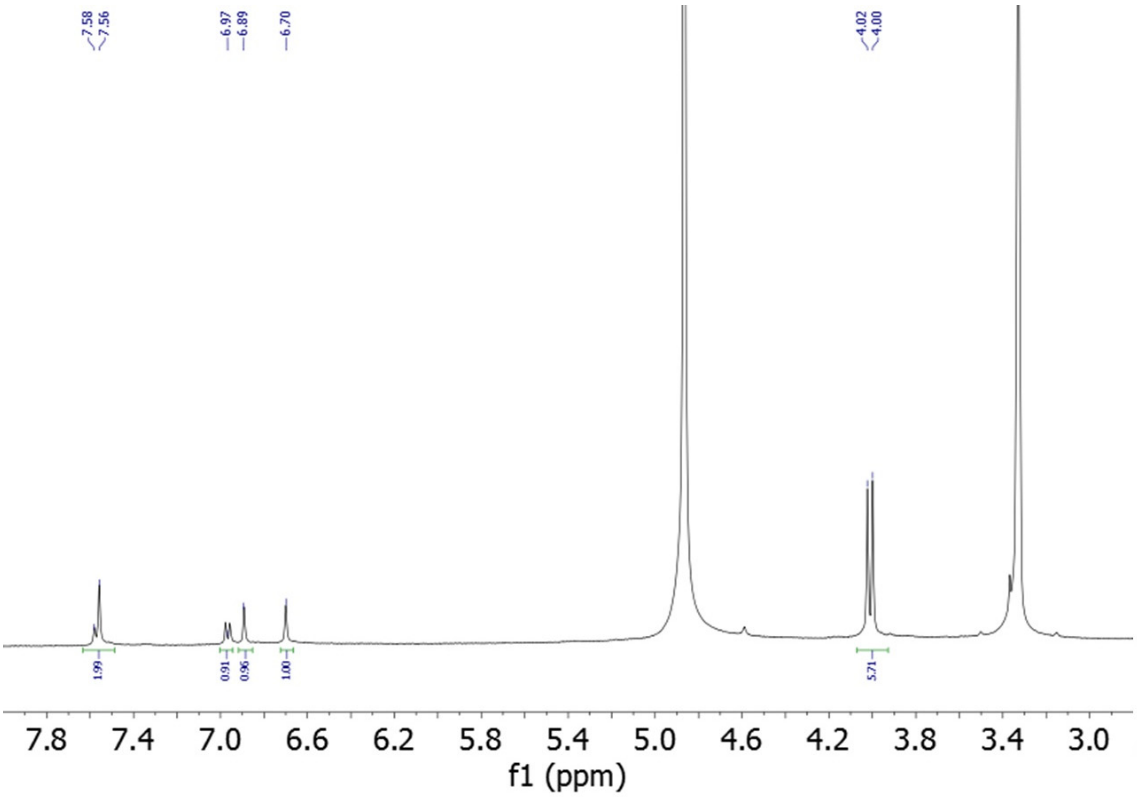

Figure S26. ^{1}H NMR Spectrum (600 MHz, CD_3OD) of compound **27**.

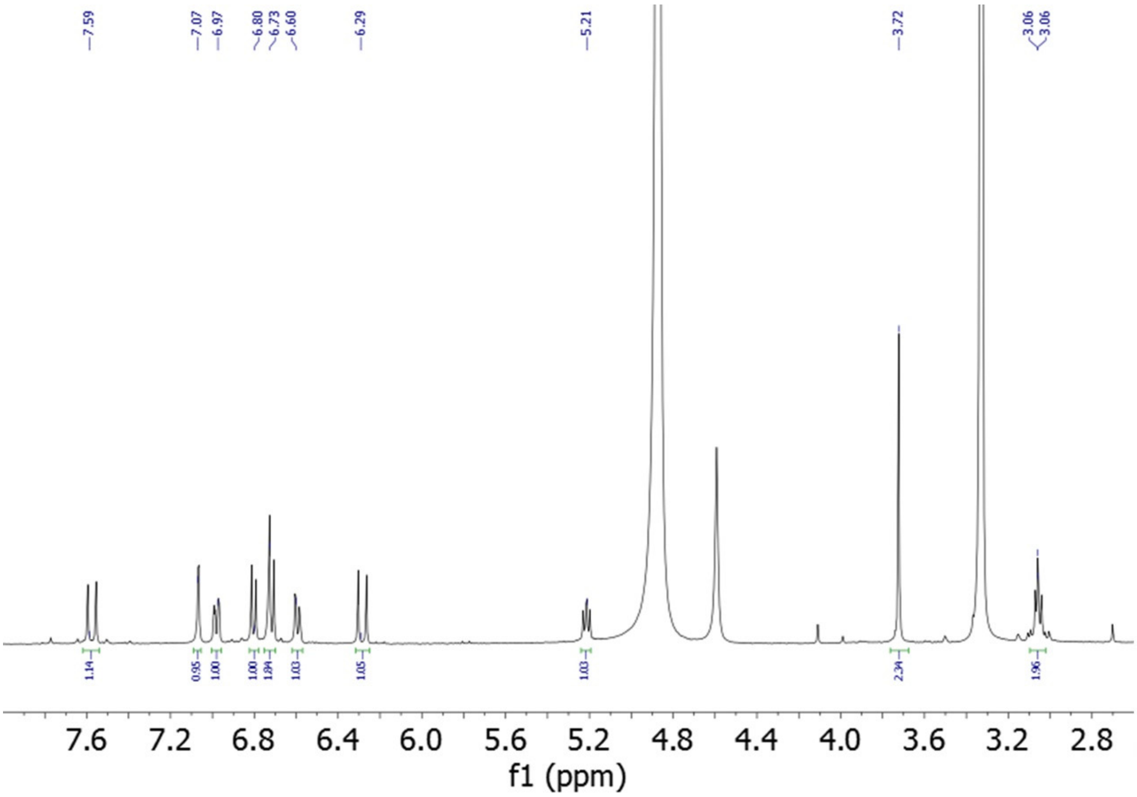

Figure S27. ^{1}H NMR Spectrum (600 MHz, CD_3OD) of compound **28**.

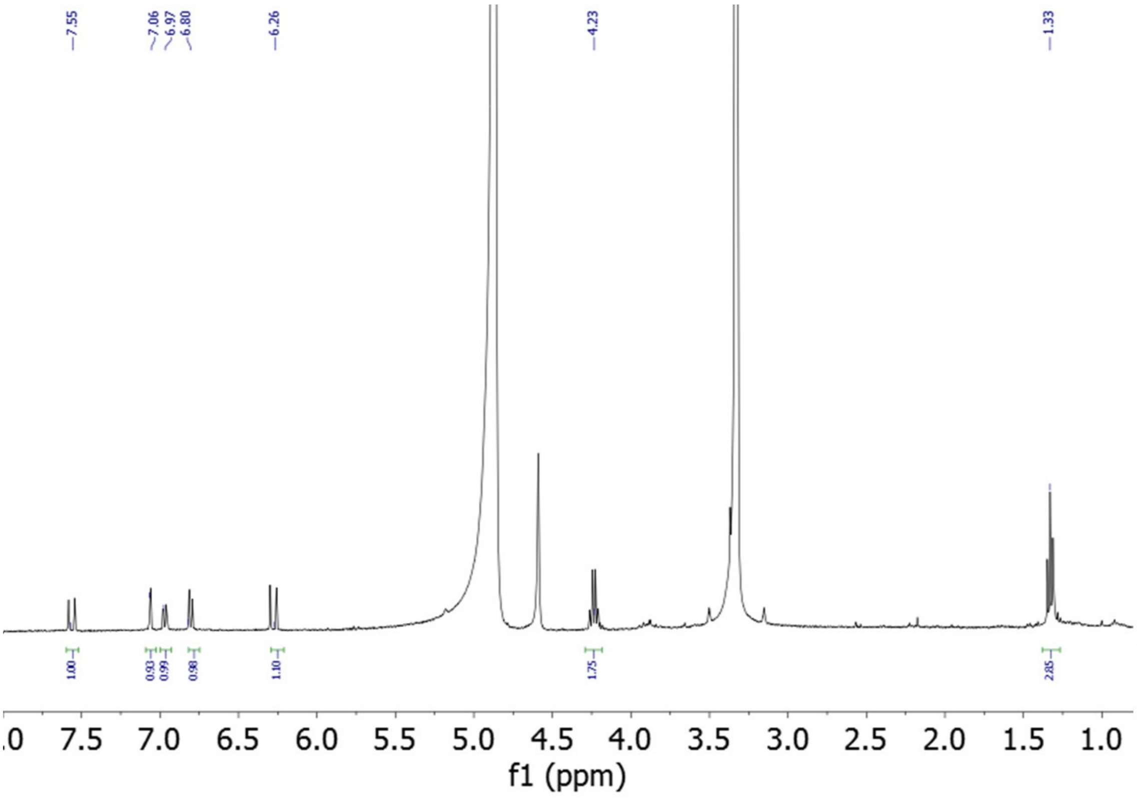

Figure S28. ^{1}H NMR Spectrum (600 MHz, CD_3OD) of compound **29**.

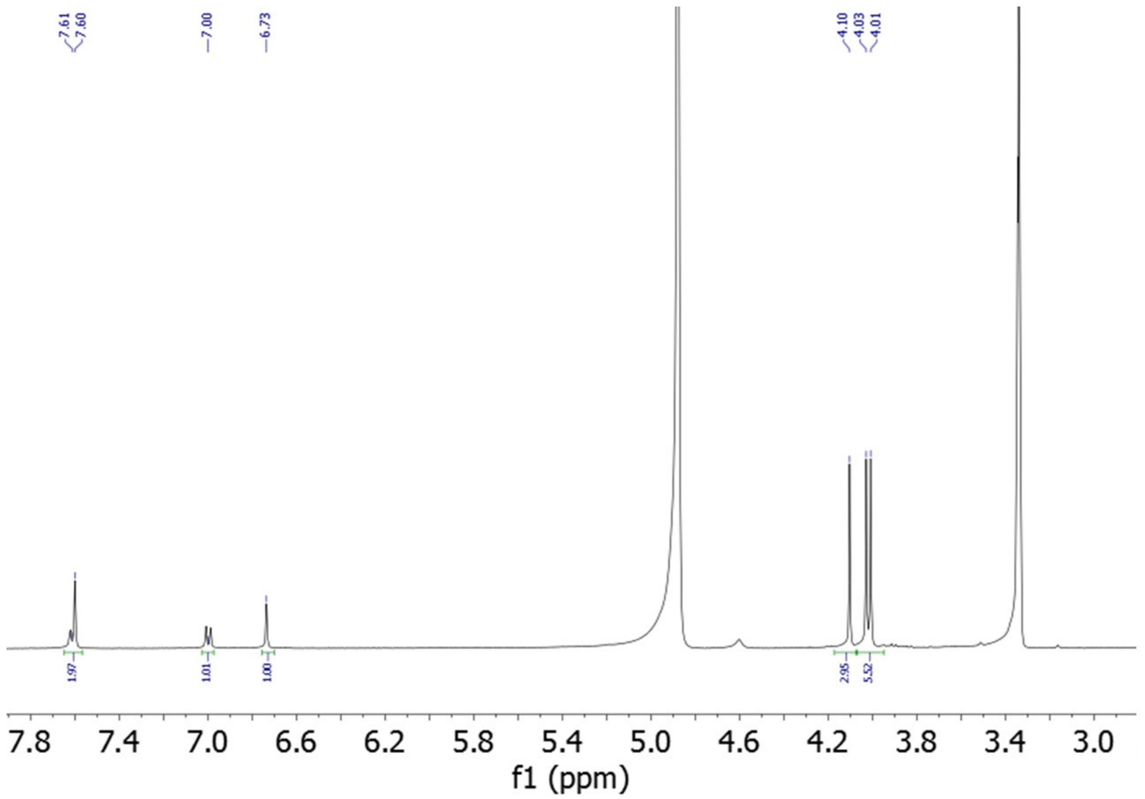

Figure S29. ^{1}H NMR Spectrum (600 MHz, CD_3OD) of compound **30**.

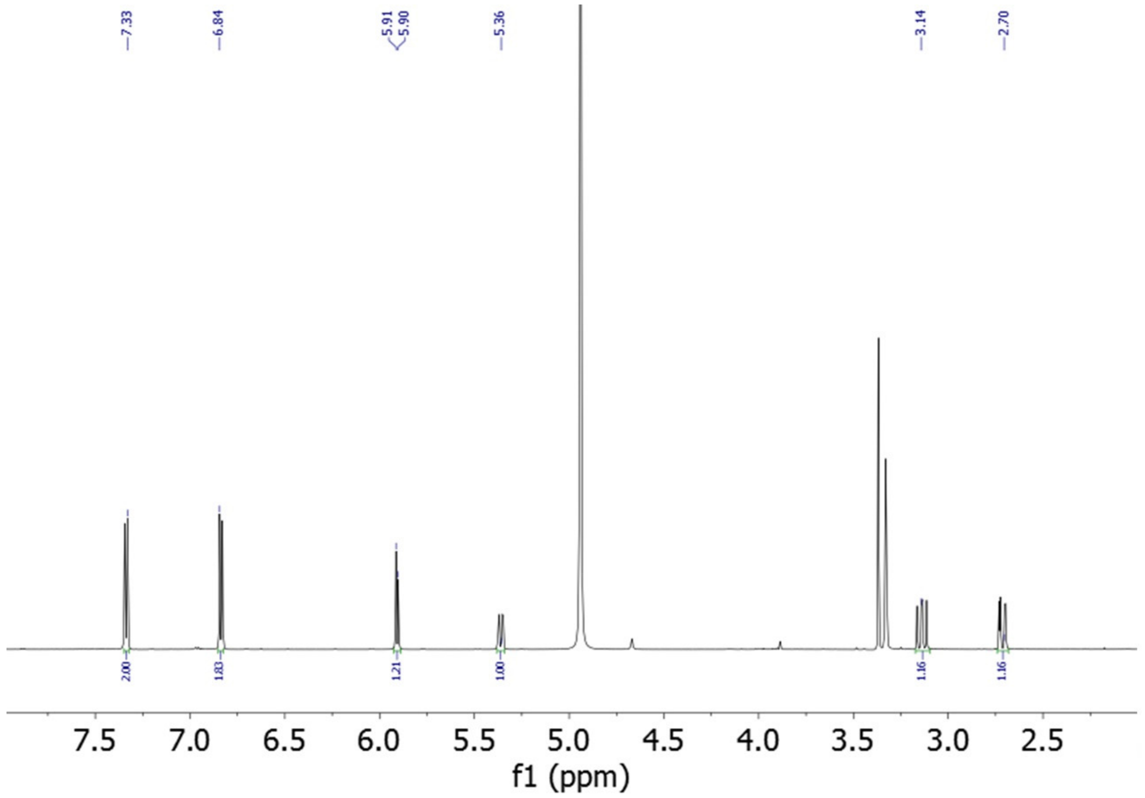

Figure S30. ^{1}H NMR Spectrum (600 MHz, CD_3OD) of compound **32**.

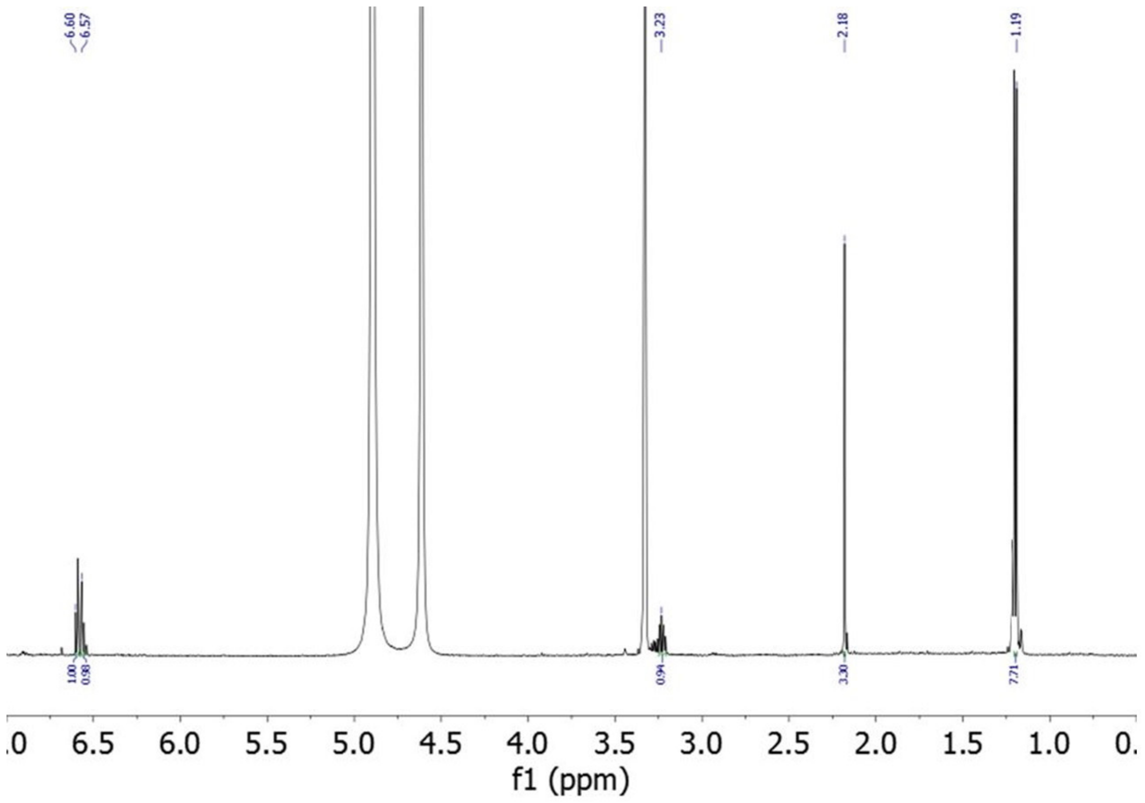

Figure S31. ^{1}H NMR Spectrum (600 MHz, CD_3OD) of compound **33**.

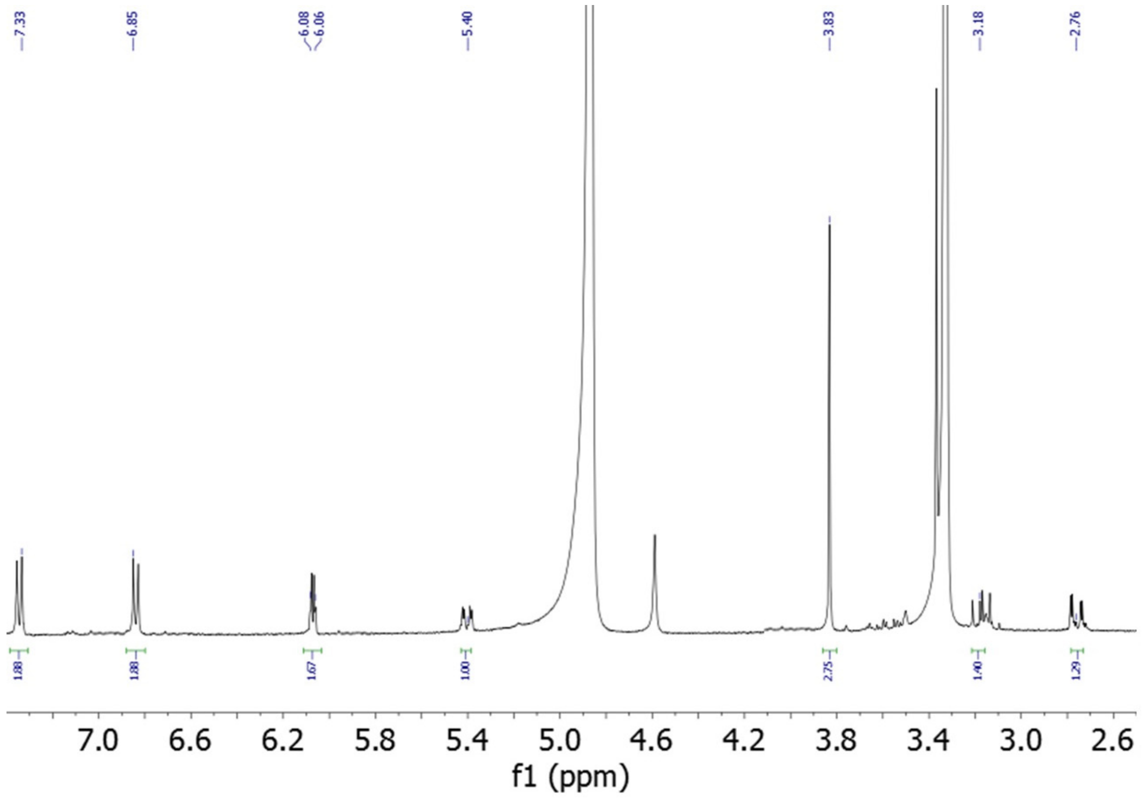

Figure S32. ^{1}H NMR Spectrum (600 MHz, CD_3OD) of compound **34**.

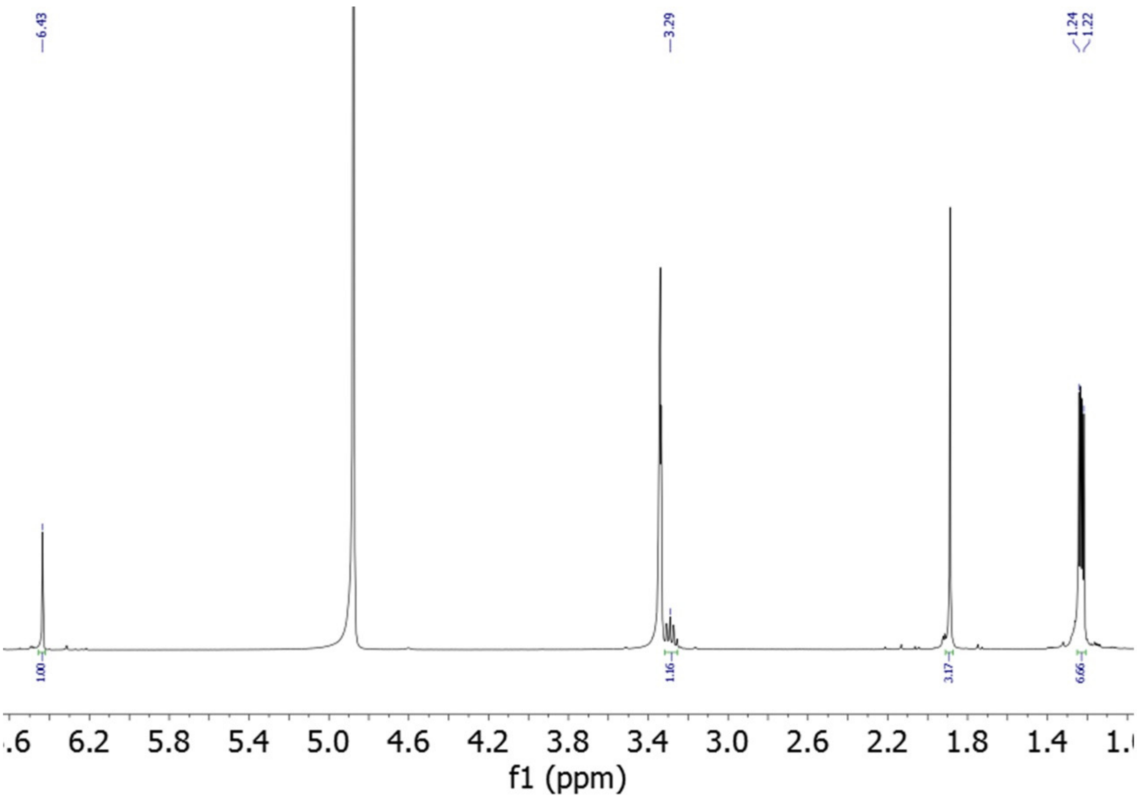

Figure S33. ^{1}H NMR Spectrum (600 MHz, CD_3OD) of compound **35**.

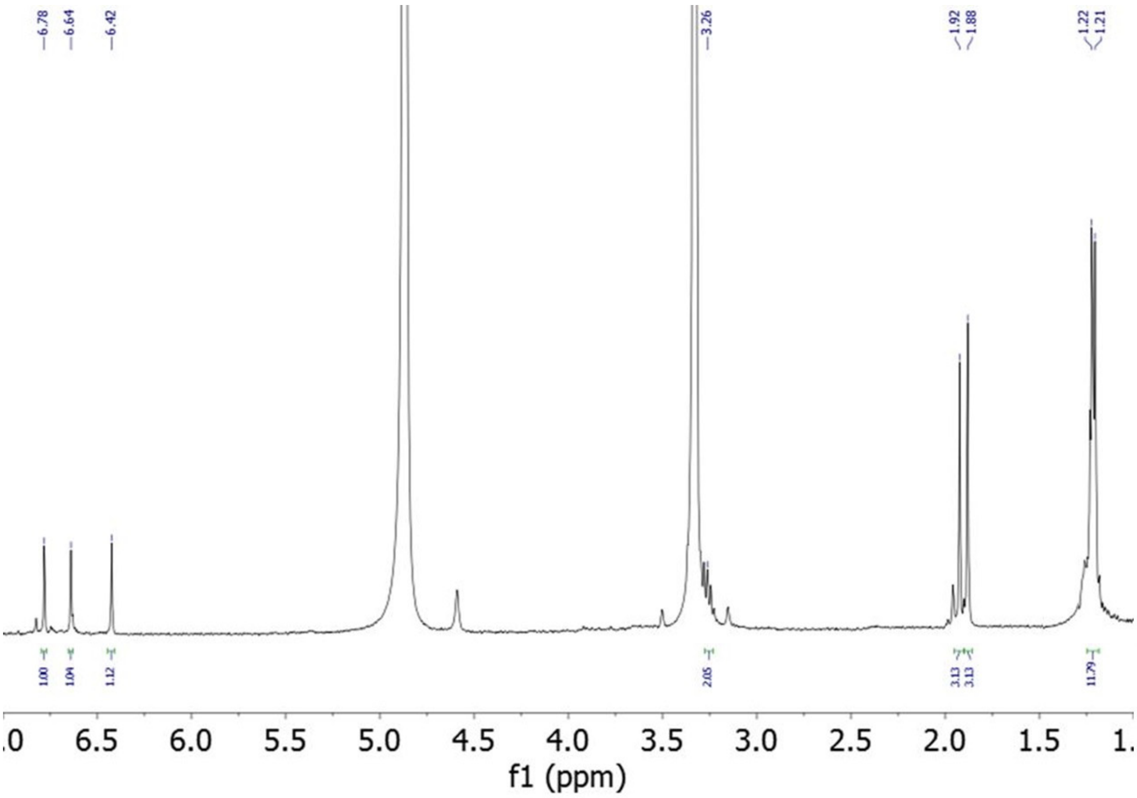

Figure S34. ^{1}H NMR Spectrum (600 MHz, CD_3OD) of compound **36**.

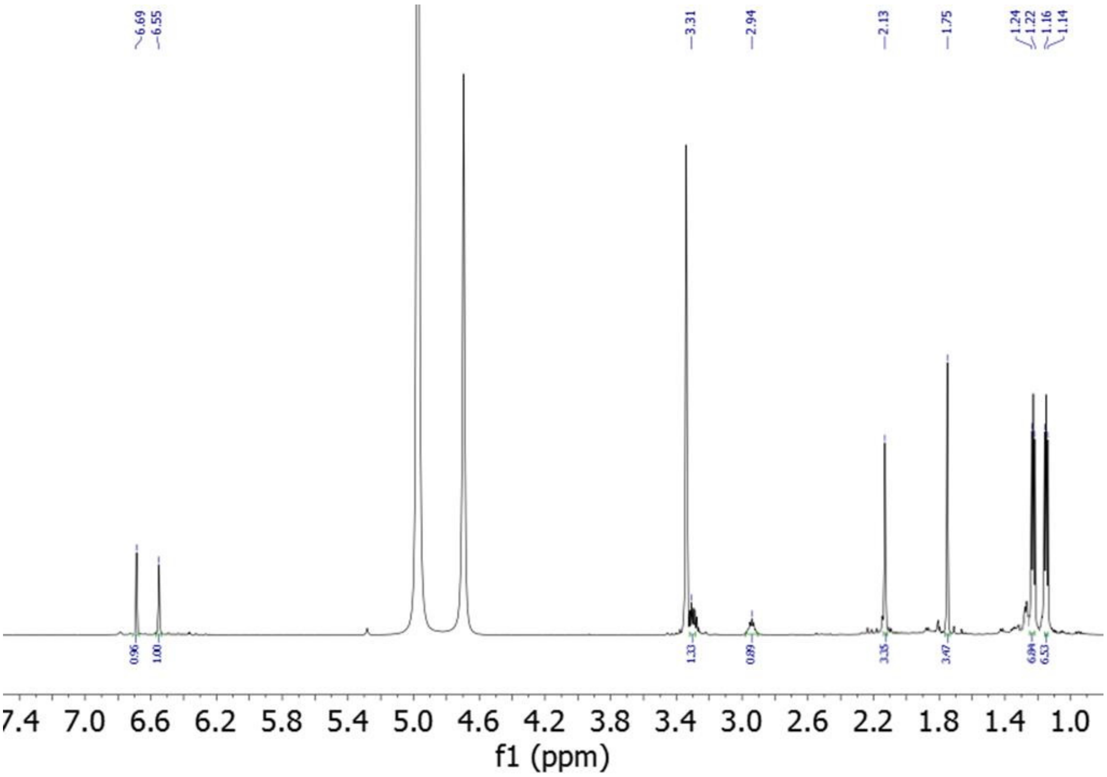

Figure S35. ^{1}H NMR Spectrum (600 MHz, CD_3OD) of compound **38**.

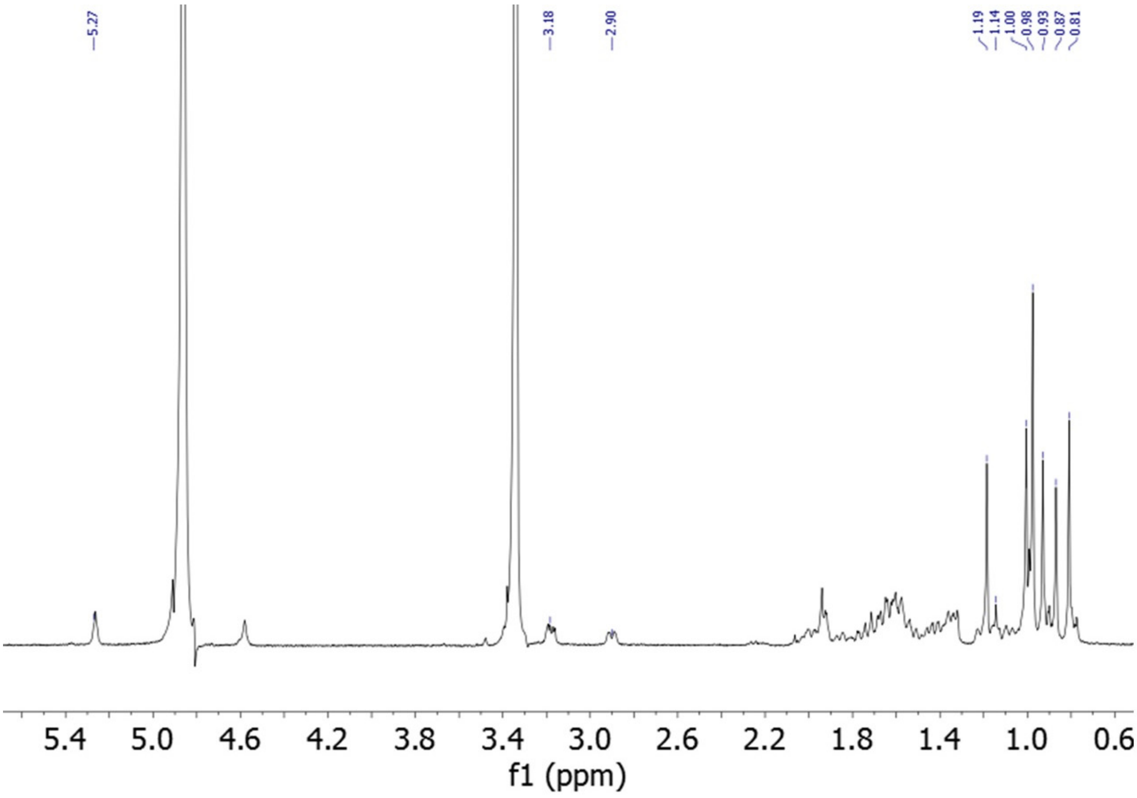

Figure S36. ^{1}H NMR Spectrum (600 MHz, CD_3OD) of compound **39**.
